# Increased habitat segregation at the dawn of the Phanerozoic revealed by correspondence analysis of bioturbation

**DOI:** 10.1038/s41598-023-49716-8

**Published:** 2023-12-15

**Authors:** Dean M. Meek, Luis A. Buatois, M. Gabriela Mángano, Bruce M. Eglington

**Affiliations:** Department of Geological Sciences, University of Saskatchewan, Saskatoon, SK S7N 5E2 Canada

**Keywords:** Palaeoecology, Palaeontology

## Abstract

The Agronomic Revolution of the early Cambrian refers to the most significant re-structuration of the benthic marine ecosystem in life history. Using a global compilation of trace-fossil records across the Ediacaran-Cambrian transition, this paper investigates the relationship between the benthos and depositional environments prior to, during, and after the Agronomic Revolution to shed light on habitat segregation via correspondence analysis. The results of this analysis characterize Ediacaran mobile benthic bilaterians as facies-crossing and opportunistic, with low levels of habitat specialization. In contrast, the Terreneuvian and Cambrian Series 2 reveal progressive habitat segregation, parallel to matground environmental restriction. This event was conducive to the establishment of distinct endobenthic communities along the marine depositional profile, showing that the increase in styles of animal-substrate interactions was expressed by both alpha and beta ichnodiversity. Habitat segregation at the dawn of the Phanerozoic may illustrate an early extension of the trophic group amensalism at community scale.

The Agronomic Revolution refers to the change in seafloor ecology from globally extensive matground environments during the Ediacaran to bioturbated mixgrounds in the Phanerozoic^1–4^. This shift was the result of increasing penetrative bioturbation, which was still rather limited during the Fortunian^5–11^ and became more widespread in shallow-marine settings during Cambrian Age 2^4,6,12,13^. These observations are supported by minor primary fabric disturbance^3,6,14^, the lack of deep-tier dwelling structures^15,16^, and geochemical proxies indicative of limited bioturbation^17,18^ in Fortunian deposits. In contrast, younger lower Cambrian deposits show a remarkable increase in ichnodiversity, modes of animal-sediment interactions, and depth and extent of bioturbation^2,3,6,12,19,20^, see^4^ for discussion.

Reconstructing spatial and temporal variation of diversity at different scales across the Ediacaran-Cambrian transition may provide clues to evaluating the mechanisms that promoted this macroecological benthic re-structuration. Diversity partitioning analysis (i.e., decomposing biodiversity into environmental and geographic components) involving comprehensive body-fossil datasets has enlightened our understanding of the ecological and evolutionary dynamics behind the Cambrian explosion^21^. However, a quantitative approach investigating questions related to diversity partitioning across the Ediacaran-Cambrian transition and its potential links to the Agronomic Revolution have not been explored from the perspective of the trace-fossil record to the same degree.

In spite of the importance of this macroecological and macroevolutionary breakthrough in the style of animal-substrate interactions, there are still few global quantitative studies revealing such changes (e.g.,^3,12,22^). We use a comprehensive ichnologic dataset from around the globe that spans the Ediacaran to early Cambrian in conjunction with correspondence analysis (CA) to observe relationships (or lack thereof) between trace-fossil occurrence and depositional environment. Trace fossils are particularly well suited for such analysis because, as the record of animal-substrate interactions, they show a direct relationship with depositional conditions and environments. As a result, trace-fossil analysis provides insights into ichnodiversity at various scales, particularly within-community (alpha ichnodiversity) and between-community (beta ichnodiversity) along the marine depositional profile (i.e., from shore to deep sea)^23^.

The issue of potential facies controls in trace-fossil distribution through Ediacaran-Cambrian sections has been repeatedly raised (e.g.,^24^). Accordingly, differentiating between facies and evolutionary controls is of paramount importance^25–27^. The quantified approach utilized displays the progressive shift from matground-dominated seafloor ecologies to modern mixground-dominated seafloor ecologies and provides evidence of habitat segregation resulting from the Agronomic Revolution.

## Results

### Ediacaran correspondence analysis

The Ediacaran dataset is the smallest of the three compiled for analysis, consisting of 277 occurrences at ichnogenus level from around the globe. In preparing the data for CA, this number was reduced further to 143 CA-compatible records (see Methods). The small sample size is attributed to the uncommon occurrence of trace fossils, likely related to the limited motility of the Ediacaran biotas^28^. Although earlier ichnologic compilations included a relatively large variety of trace fossils for the Ediacaran^29,30^, subsequent work has re-interpreted many of these as body fossils or microbially induced sedimentary structures, therefore significantly reducing ichnodiversity levels^3,8,9,19,28,31,32^.

**Figure 1:**
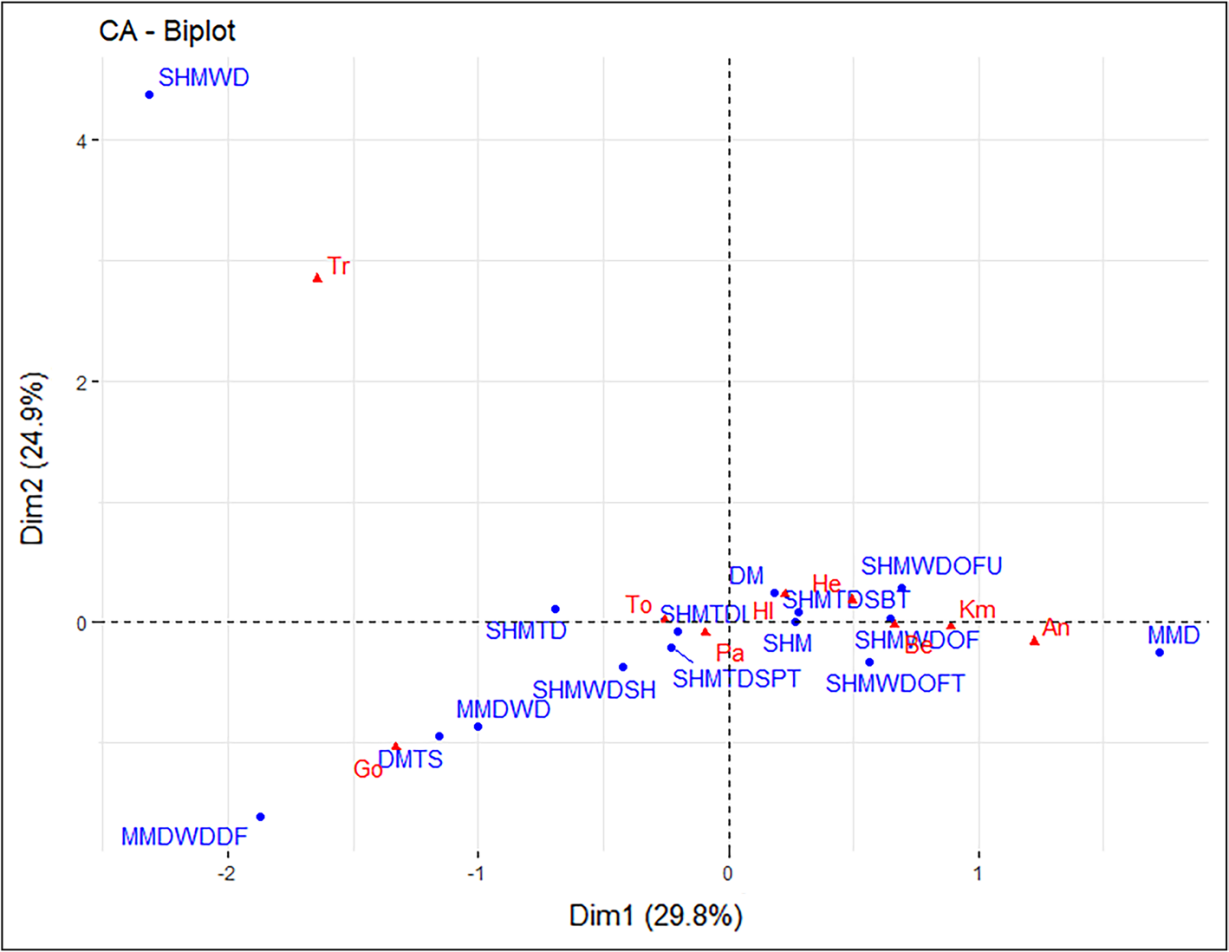
Ediacaran correspondence analysis biplot, displaying the relationship between the observations (depositional environments—blue circles) and variables (ichnogenera—red triangles). Plot created in R with the factoextra package^34^. Abbreviations are as follows: *Archaeonassa* (An), *Bergaueria* (Be), *Gordia* (Go), *Helminthoidichnites* (He), *Helminthopsis* (Hl), *Kimberichnus* (Km), *Palaeophycus* (Pa), *Torrowangea* (To), treptichnids (Tr); Deep marine (DM), Deep marine turbidite system (DMTS), Marginal marine deltaic (MMD), Marginal marine deltaic—wave dominated (MMDWD), Marginal marine deltaic—wave dominated delta front (MMDWDDF), Shallow marine (SHM), Shallow marine tide dominated (SHMTD), Shallow marine tide dominated—Intertidal (SHMTDI), Shallow marine tide dominated—supratidal (SHMTDSPT), Shallow marine wave dominated (SHMWD), Shallow marine wave dominated – offshore (SHMWDOF), Shallow marine wave dominated—offshore transition (SHMWDOFT), Shallow marine wave dominated—offshore transition (SHMWDOFT), and Shallow marine wave dominated—shelf (SHMWDSH).

**Figure 2:**
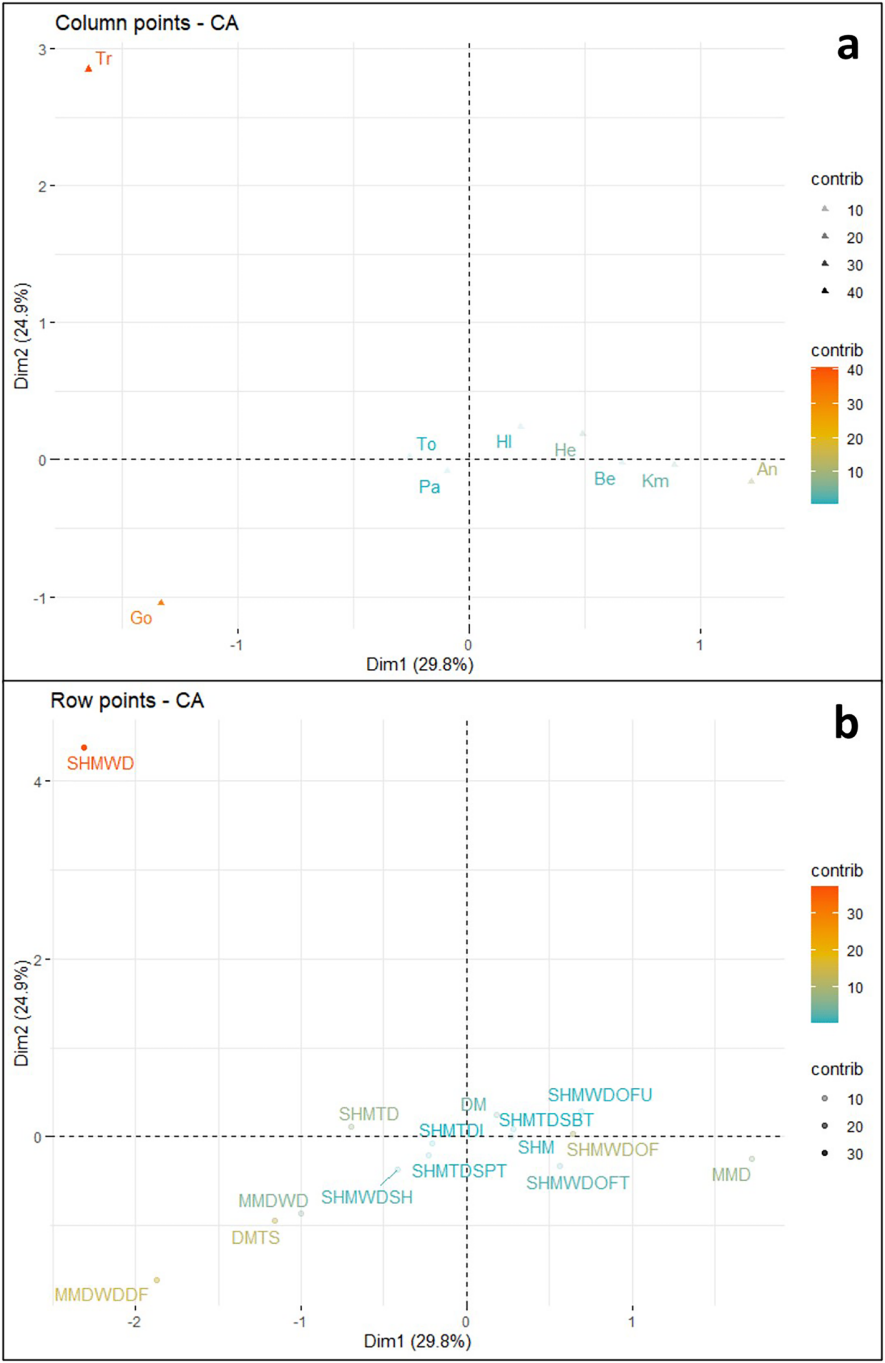
(**a**) Visualization of Ediacaran column variable contribution towards dimensions 1 and 2 of the CA. (**b**) Visualization of Ediacaran row observation contribution towards dimensions 1 and 2 of the CA. Both plots created in R with the factoextra package^34^.

All CA compatible records were compiled into a contingency table in preparation for analysis. A Chi-square test (see R Code in supplementary material) indicates a statistical significance between rows and columns in this dataset, supporting the use of CA. Conducting analysis in R using FactoMinR^33^, the Ediacaran CA biplot (Fig. 1) displays the two largest and most significant ordination axes representing 29.8% and 24.9% of the variance within the dataset. A third axis was retained for analysis based on CA best practice A review of data point contribution and quality of representation reveals a lack of readily explainable patterns and relationships in the Ediacaran biplot. Individual observations (depositional environments) and variables (ichnogenera) contribute to the ordination dimensions in varying amounts, with larger contributions representing more importance in describing the variability within the dataset^34^. Any contribution greater than the average expected value if data were random is considered significant in these results. Dimension 1 displays several significant contributions, including a positive contribution from the *Archaeonassa* variable, a negative contribution from the treptichnid variable, and a large negative contribution from the *Gordia* variable (Fig. 2a). Significant positive contributions are displayed by SHMWDOF and MMD observations for dimension 1, while significant negative contributions are displayed by SHMWD, MMDWDDF, DMTS, and SHMTD observations (Fig. 2b). Key contributors towards dimension 2 in the ordination include a large positive contribution from the treptichnid variable, and a negative contribution from the *Gordia* variable. Important contributions from observations are a large positive contribution from SHMWD, and negative contributions from MMDWDDF and DMTS observations.

**Figure 3:**
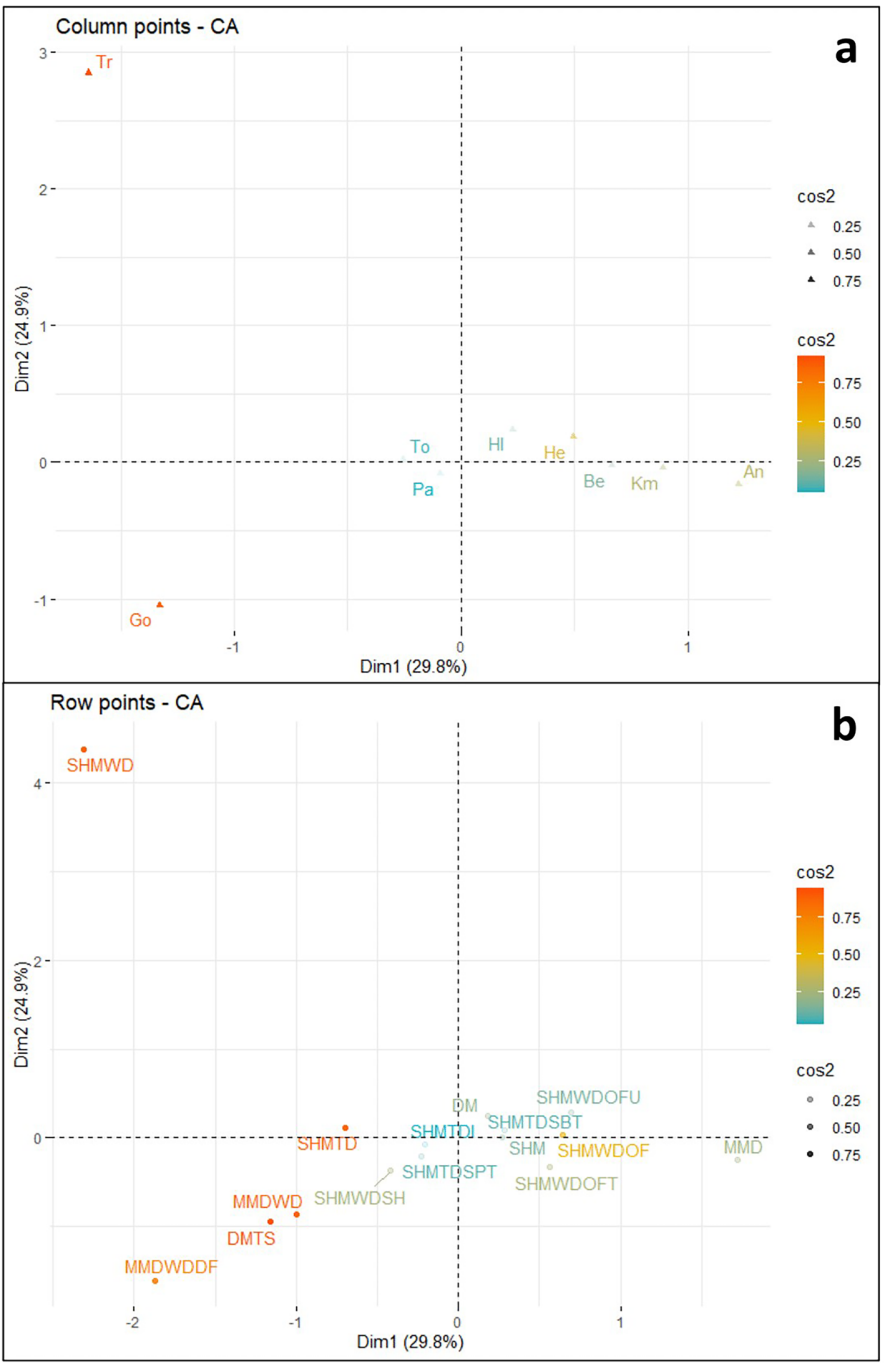
(**a**) Visualization of Ediacaran variable quality of representation in dimensions 1 and 2 of the CA. (**b**) Visualization of Ediacaran observation quality of representation in dimensions 1 and 2 of the CA. Both plots created in R with the factoextra package^34^.

A second aspect to consider when interpreting biplots is the quality of representation for data points, described by the squared cosine (cos^2^) metric. This metric indicates if a point is well represented by two dimensions^34^, with a value of 1 indicating the point is well represented and falls within the plane of the ordination axes, and a value of 0 indicating that the points are poorly represented and are orthogonal to the ordination axes. For the description of results, values ≥ 0.66 are considered well represented, and values ≥ 0.33 are considered moderately represented. The treptichnid and *Gordia* variables are well represented by the first two ordination axes (Fig. 3a). Quality of representation drops significantly after the first two variables, with *Helminthoidichnites* displaying moderate representation, and *Archaeonassa*, *Kimberichnus, Bergaueria*, *Helminthopsis*, *Torrowangea*, and *Palaeophycus* poorly represented by the first two axes (see supplementary material Fig. S6). The DMTS, SHMWD, MMDWD, SHMTD, and MMDWDDF observations are well represented by the first two ordination axes (Fig. 3b). Quality of representation drops slightly to moderate for the SHMWDOF observation, followed by a large drop with all remaining observations poorly represented by the first two ordination axes.

Evaluating the resulting biplot relies on the identification of variables and observations that are in close proximity to each other^35^. Distance between observation and variable points cannot be directly compared; rather, the important factor is the angular distance between points^34^. If the points plotted had a line drawn back to the origin of the biplot, an acute angle between a variable and an observation indicates a strong association^34^, whereas a right angle would indicate no association and an obtuse angle indicates negative association. Component 1 of the Ediacaran biplot (Fig. 2) explains the largest amount of variance in the dataset, accounting for 29.8%. The most significant variables and observations to consider in the interpretation are those that display large contributions to the ordination and are well summarized in the axes displayed as previously described. The treptichnid and *Gordia* variables show strong contributions and are well summarized in the first two ordination axes, while the *Archaeonassa* variable shows a strong contribution, but it is not well summarized in the first two ordination axes. These variables are plotted in close proximity to disparate depositional environments (e.g., shallow-marine, deep-marine, and marginal-marine), lacking any clear or meaningful associations. The original input data (i.e., the contingency Table S2 found in supplementary material) helps to illustrate the above observations. For example, *Gordia* and *Archaeonassa* are rarely observed in the same depositional environment and thus are found on opposing ends of component 1.

Component 2 of the Ediacaran biplot represents 24.9% of the variance within the dataset. The treptichnid and *Gordia* variables display the largest contributions to this component. In this biplot the magnitude (i.e., distance from the origin) of the treptichnid variable is the most distinct feature. This should be viewed with caution, as variables with very few occurrences can be pushed to the periphery of the biplot in CA leading to a false sense of importance^36^. Rather than focusing on the linear distance from other points, the angular distance from other variables (i.e., ichnogenera) suggests this is a unique and distinct variable. In addition, it should be considered that Ediacaran forms included herein as treptichnids are restricted to siliciclastic deposits of the terminal Ediacaran (i.e., Nama assemblage) and have not been recorded in older Ediacaran rocks^37–39^. An additional axis (i.e., component 3) resulting from the Ediacaran CA was retained and analyzed, this component did not contain discernable patterns between or within the variables and observations (see supplementary material Fig. S10).

### Terreneuvian correspondence analysis

The trace-fossil dataset for the Terreneuvian is significantly larger than that of the Ediacaran, consisting of 750 trace-fossil records. Following the identification of CA-compatible records (see Methods), a total of 521 entries were used in this analysis. The large increase in sample size across the Ediacaran to Cambrian transition is not unexpected, as previous quantitative analyses have demonstrated an increased trace-fossil broad morphologic diversity (ichnodisparity), behavioral complexity, and a more efficient endobenthic ecospace utilization^3^.

**Figure 4:**
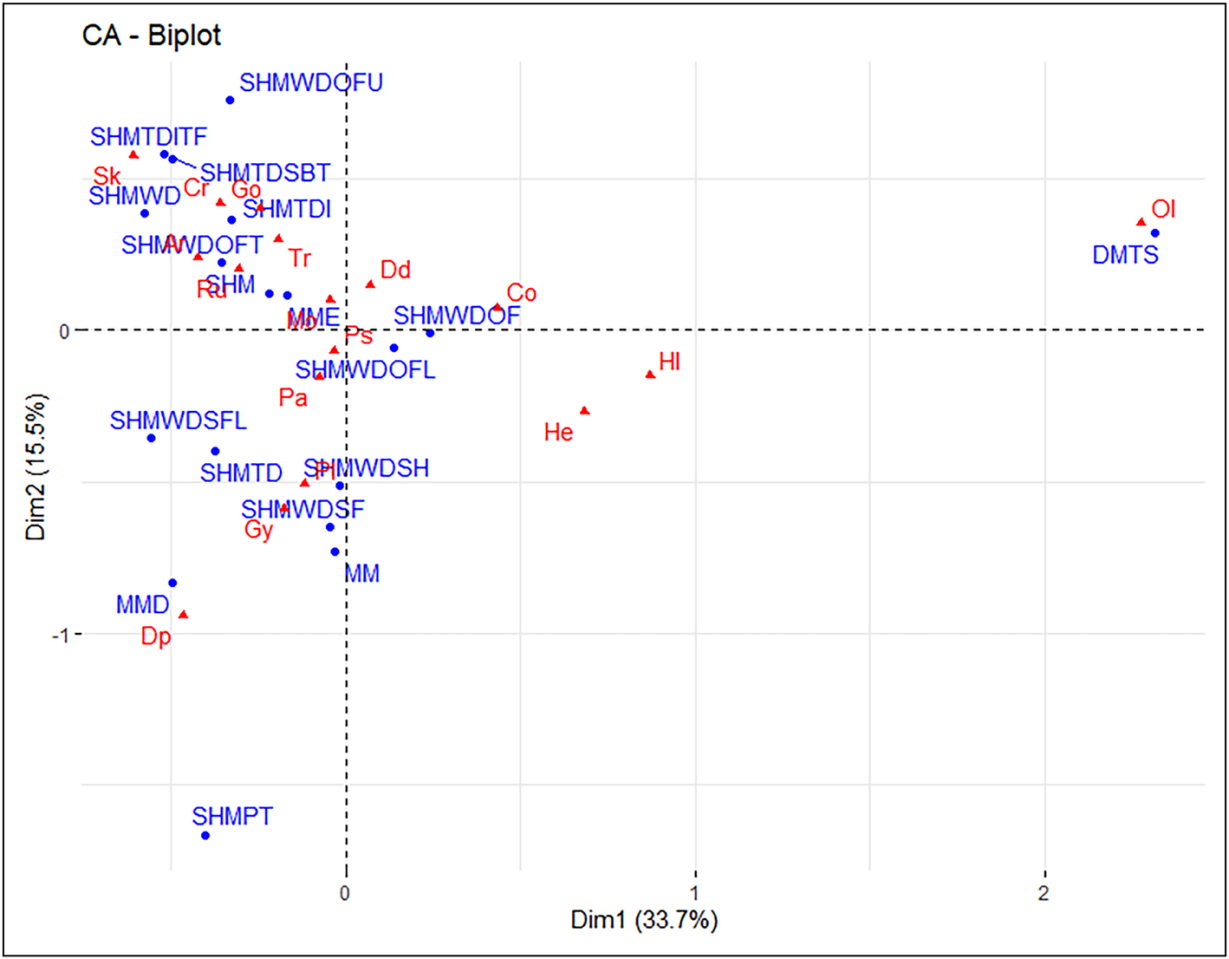
Terreneuvian correspondence analysis biplot, displaying the relationship between the observations (depositional environments—blue circles) and variables (ichnogenera—red triangles). Plot created in R with the factoextra package^34^. Abbreviations are as follows: *Arenicolites* (Ar), *Cochlichnus* (Co), *Cruziana* (Cu), *Didymaulichnus* (Dd), *Diplocraterion* (Dp), *Gordia* (Go), *Gyrolithes* (Gy), *Helminthoidichnites* (He), *Helminthopsis* (Hl), *Monomorphichnus* (Mo), *Oldhamia* (Ol), *Palaeophycus* (Pa), *Planolites* (Pl), *Psammichnites* (Ps), *Rusophycus* (Ru), *Skolithos* (Sk), and *Treptichnus* (Tr); Deep marine turbidite system (DMTS), Marginal marine (MM), Marginal marine estuary (MME), Marginal marine deltaic (MMD), Shallow marine (SHM), Shallow marine platform (SHMPT), Shallow marine tide dominated (SHMTD), Shallow marine tide dominated—intertidal (SHMTDI), Shallow marine tide dominated—intertidal tidal flat (SHMTDITF), Shallow marine tide dominated—subtidal (SHMTDSBT), Shallow marine wave dominated (SHMWD), Shallow marine wave dominated – shoreface (SHMWDSF), Shallow marine wave dominated—shoreface lower (SHMWDSFL), Shallow marine wave dominated—offshore (SHMWDOF), Shallow marine wave dominated—offshore lower (SHMWDOFL), Shallow marine wave dominated – offshore transition (SHMWDOFT), Shallow marine wave dominated—offshore upper (SHMWDOFU), and Shallow marine wave dominated—shelf (SHMWDSH).

Correspondence Analysis was conducted on the Terreneuvian dataset, and a biplot was created displaying the two most significant ordination axes (Fig. 4) representing nearly 50% of the variance. Ordination axes three and four were retained (see supplementary material Fig. S20) for analysis based on CA best practices^34,40^.

**Figure 5:**
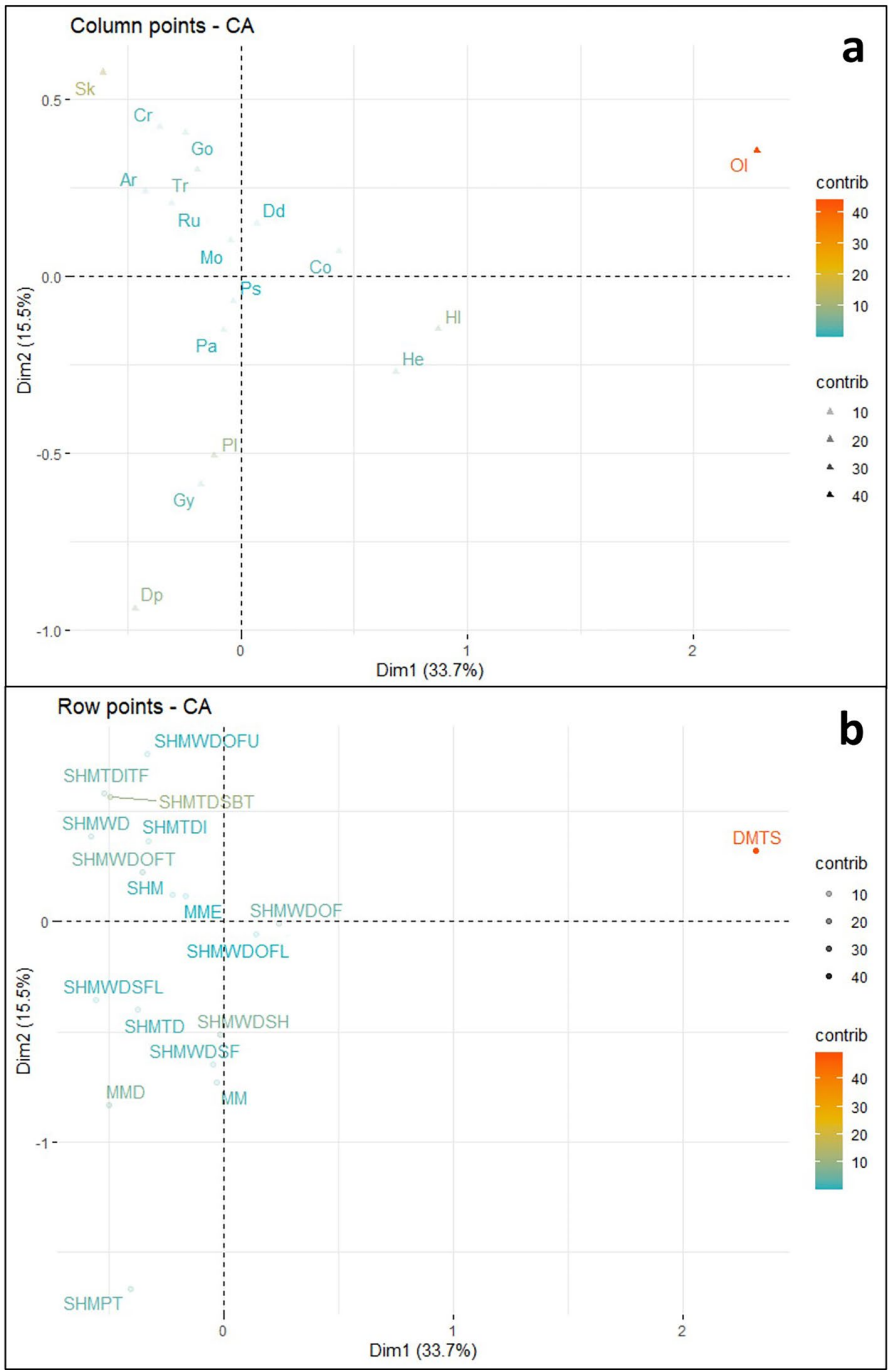
(**a**) Visualization of Terreneuvian column variable contribution towards dimensions 1 and 2 of the CA. (**b**) Visualization of Terreneuvian row observation contribution towards dimensions 1 and 2 of the CA. Both plots created in R with the factoextra package^34^.

Data point contributions for dimension 1 in the Terreneuvian biplot were dominated by the positive contribution from the *Oldhamia* variable (Fig. 5a) and the positive contribution from the DMTS observation (Fig. 5b). Dimension 1 also displays a smaller positive contribution from the *Helminthopsis* variable, and smaller negative contributions from the *Skolithos* variable and the SHMTDSBT observation. The original input data (i.e., the contingency Table S3 found in supplementary material) supports these observations, with *Oldhamia* far outnumbering other ichnogenera in the DMTS depositional environment leading to a distinction on dimension 1. Further, *Oldhamia* is rarely found in the same depositional environment as ichnogenera that plot on the negative side of dimension 1, such as *Skolithos* and *Diplocraterion*.

Significant positive contributions in dimension 2 came from the *Skolithos*, and *Treptichnus* variables, and the SHMTDSBT, and SHMTDITF observations. Significant negative contributions in dimension 2 came from the *Planolites*, *Diplocraterion*, and *Gyrolithes* variables, and the SHMWDSH, MMD, SHMPT, and MM observations. Again, the original input data provides some insight into these observations, with *Skolithos* outnumbering other ichnogenera in the SHMTDSBT depositional environment, and *Planolites* outnumbering other ichnogenera in the SHMWDSH depositional environment.

**Figure 6:**
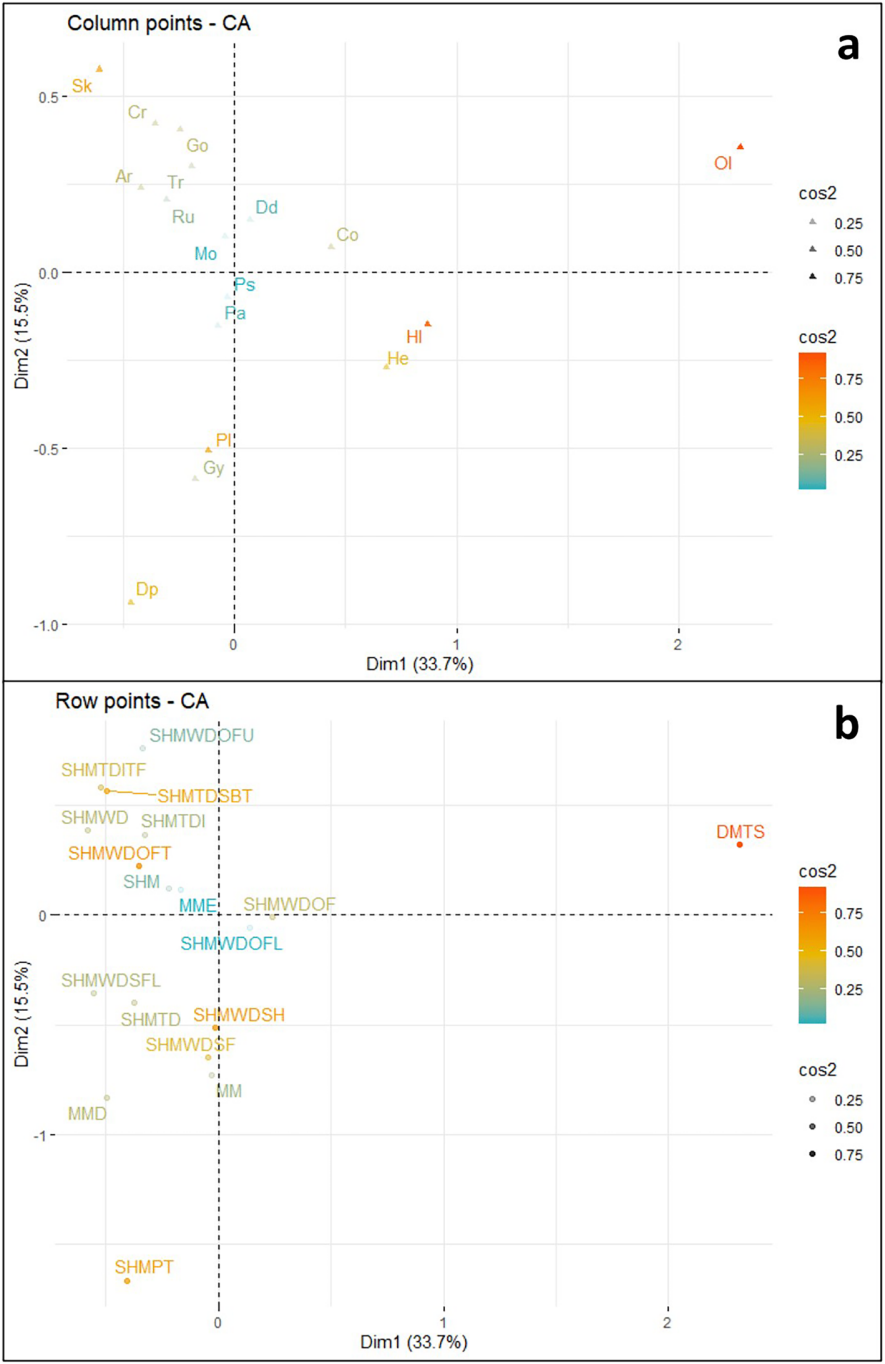
(**a**) Visualization of Terreneuvian variable quality of representation in dimensions 1 and 2 of the CA. (**b**) Visualization of Terreneuvian observation quality of representation in dimensions 1 and 2 of the CA. Both plots created in R with the factoextra package^34^.

Data point quality of representation for the first two dimensions in the Terreneuvian biplot indicates that the *Oldhamia*, and *Helminthopsis* variables are well represented by the first two ordination axes (Fig. 6a). Additional variables show a decrease in the quality of representation, with *Planolites*, *Skolithos, Diplocraterion,* and *Helminthoidichnites* showing moderate representation by dimensions 1 and 2. Among the observations (Fig. 6b), DMTS displays the highest quality of representation. This is followed by a drop, and SHMWDOFT, SHMWDSH, SHMPT, SHMTDSBT, SHMWDSF, and SHTDITF observations displaying moderate quality of representation.

### Cambrian series 2 correspondence analysis

The third and final dataset analyzed was composed of trace fossils recorded for Cambrian Series 2. The initial dataset was composed of 1686 records, which was reduced in the process of identifying CA-compatible records to 1194, making this the largest of the three datasets analyzed. Although the larger number of trace-fossil occurrences may reflect in part an increased number of trace-fossil localities, recent work showed that the increase in the number of ichnologic metrics is largely reflecting new behaviors and a substantial exploitation of the endobenthic ecospace^12^.

**Figure 7:**
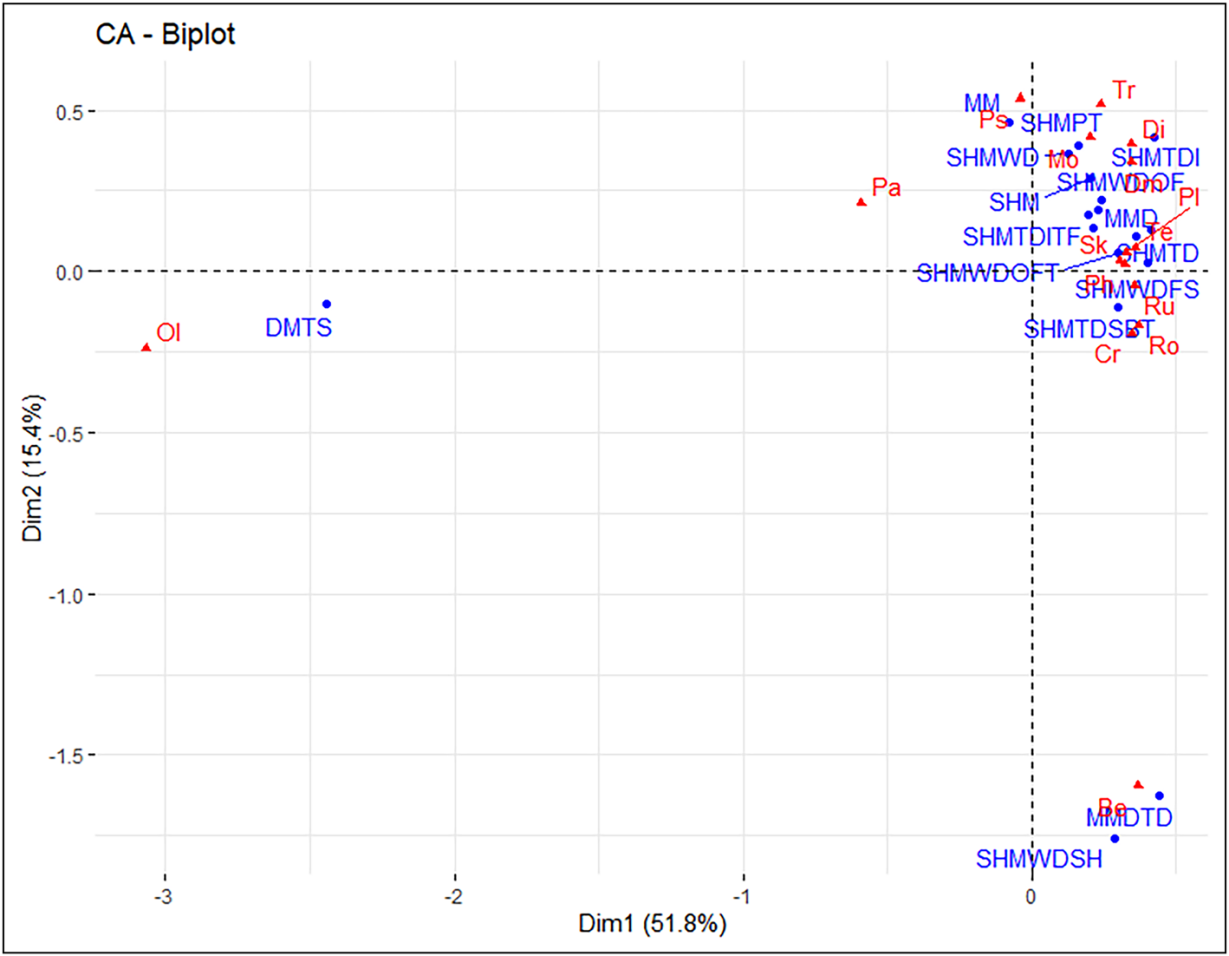
Cambrian Series 2 correspondence analysis biplot, displaying the relationship between the observations (depositional environments—blue circles) and variables (ichnogenera—red triangles). Plot created in R with the factoextra package^34^. Abbreviations are as follows: *Bergaueria* (Be), *Cruziana* (Cr), *Dimorphichnus* (Dm), *Diplichnites* (Di), *Monomorphichnus* (Mo), *Oldhamia* (Ol), *Palaeophycus* (Pa), *Phycodes* (Ph), *Planolites* (Pl), *Psammichnites* (Ps), *Rosselia* (Ro), *Rusophycus* (Ru), *Skolithos* (Sk), *Teichichnus* (Te), and *Treptichnus* (Tr); Deep marine—turbidite system (DMTS), Marginal marine (MM), Marginal marine deltaic (MMD), Marginal marine deltaic—tide dominated (MMDTD), Shallow marine (SHM), Shallow marine platform (SHMPT), Shallow marine tide dominated (SHMTD), Shallow marine tide dominated—intertidal (SHMTDI), Shallow marine tide dominated—intertidal tidal flat (SHMTDITF), Shallow marine tide dominated—subtidal (SHMTDSBT), Shallow marine wave dominated (SHMWD), Shallow marine wave dominated—foreshore (SHMWDFS), Shallow marine wave dominated—shoreface (SHMWDSF), Shallow marine wave dominated—shoreface lower (SHMWDSFL), Shallow marine wave dominated—offshore transition (SHMWDOFT), Shallow marine wave dominated—offshore (SHMWDOF), and Shallow marine wave dominated—shelf (SHMWDSH).

Conducted CA on the Cambrian Series 2 dataset, and a biplot of components 1 and 2 (Fig. 7) was created. In contrast to the biplots from the previous two periods, component 1 alone represents over 50% of the variance. Combined, the biplot represents more than 65% of the variance within the dataset. Components 3 and 4 were retained for analysis as well (see supplementary material Fig. S30).

**Figure 8:**
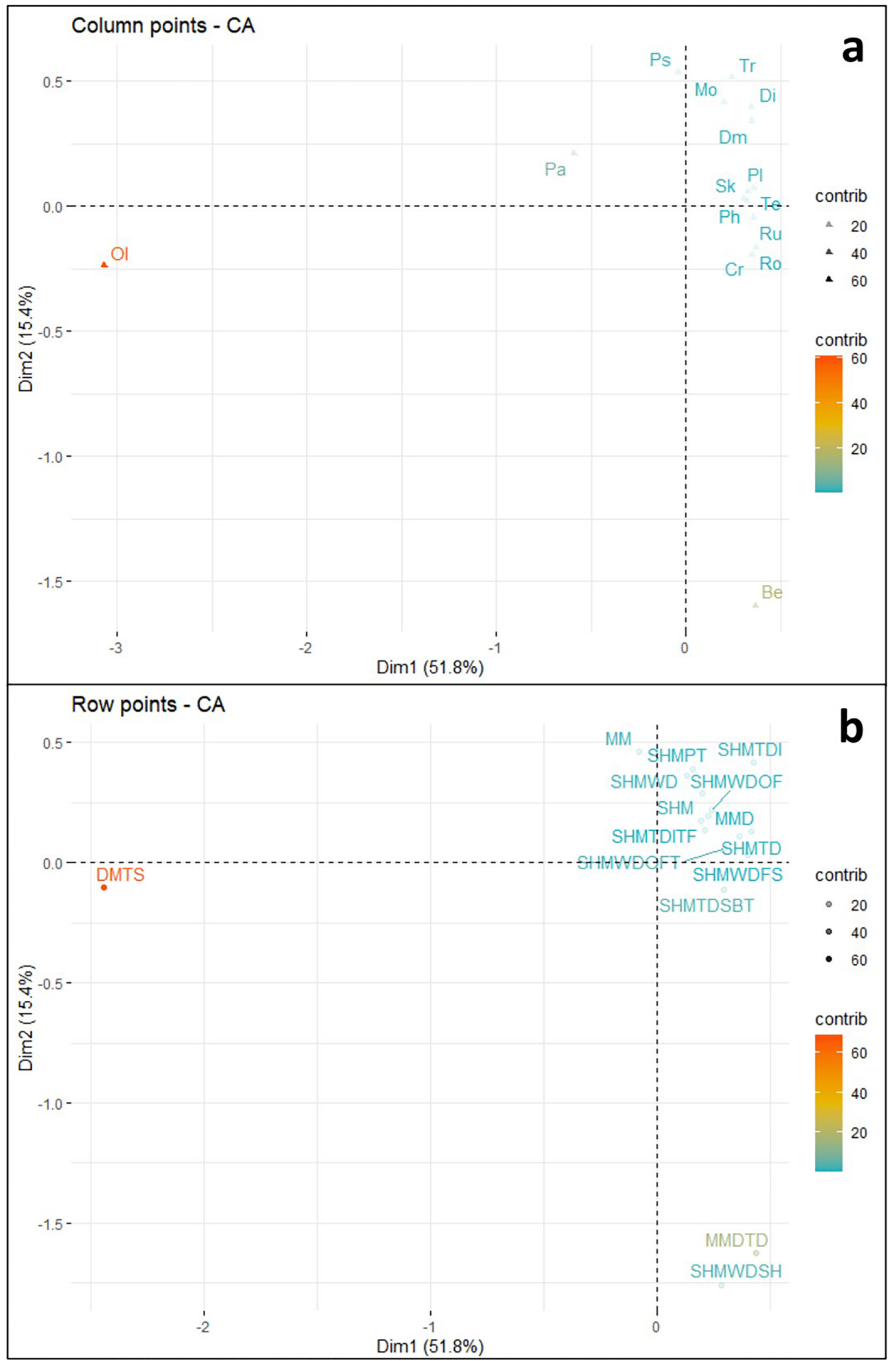
(**a**) Visualization of Cambrian Series 2 column variable contribution towards dimensions 1 and 2 of the CA. (**b**) Visualization of Cambrian Series 2 row observation contribution towards dimensions 1 and 2 of the CA. Both plots created in R with the factoextra package (Kassambara & Mundt, 2020).

Data point contributions for dimension 1 in the Cambrian Series 2 biplot were dominated by the negative contribution from the *Oldhamia* variable (Fig. 8a) and the negative contribution from the DMTS observation (Fig. 8b). *Palaeophycus* displays a small negative contribution to dimension 1. The clear distinction of both *Oldhamia* and DMTS in dimension 1 is a continuation of the pattern observed in the Terrenuvian CA. The Cambrian Series 2 biplot shows a much larger distinction between this ichnogenus and depositional environmental pair, with the original data (i.e., the contingency Table S4 found in supplementary material) showing that *Oldhamia* is found exclusively in DMTS. This is in contrast to the Terrenuvian original data, where the ichnogenus was also found in deeper shallow marine settings (e.g., SHMWDOF and SHMWDSH).

Contributions for dimension 2 were controlled largely by the negative influence of the *Bergaueria* variable and the MMDTD observation. The SHMWDSH observation displays a minor negative influence. There were no significant positive contributions from variables or observations in the first two ordination axes. Original input data shows that more than one third of the *Bergaueria* occurrences are in MMDTD and *Bergaueria* also account for half of the occurrences in SHMWDSH. The disproportionate number of this ichnogenera present in these depositional environments explains this distinction from other ichnogenera and depositional environments.

**Figure 9:**
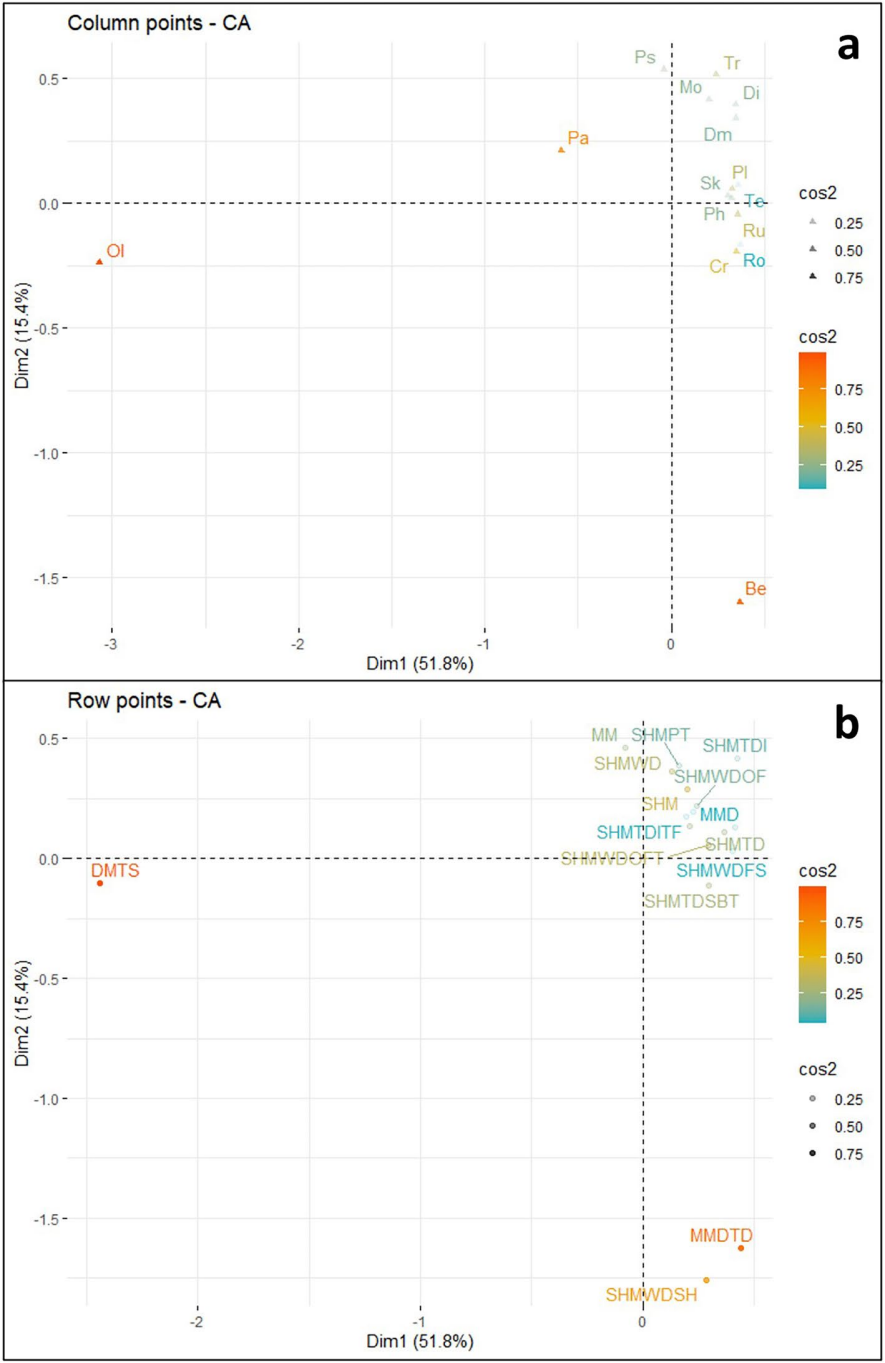
**(a**) Visualization of Cambrian Series 2 variable quality of representation in dimensions 1 and 2 of the CA. (**b**)—Visualization of Cambrian Series 2 observation quality of representation in dimensions 1 and 2 of the CA. Both plots created in R with the factoextra package (Kassambara & Mundt, 2020).

Data point quality of representation for dimensions one and two in the Cambrian Series 2 biplot indicates that the *Oldhamia*, *Bergaueria,* and *Palaeophycus* variables are well represented by the first two ordination axes (Fig. 9a). Additional variables with moderate representation by dimensions 1 and 2 include *Cruziana*, *Rusophycus*, *Planolites*, and *Treptichnus*. Observations that display a high quality of representation in the biplot includes DMTS, MMDTD, and SHMWDSH (Fig. 9b). Quality of representation drops significantly after the previously mentioned observations, with the SHM and SHMWDOFT observations showing moderate representation.

## Discussion

Correspondence Analysis reveals a structure in the analyzed datasets that promotes the identification of correlations between variables (ichnogenera) and relationships between variables and observations (depositional environments). This type of numerical analysis represents a tool to unravel diversity partitioning and facilitate analysis to determine how it relates to benthic macroecological breakthroughs across the Ediacaran-Cambrian transition. In addition, a new quantitative exploration of the data allows testing previously proposed hypotheses and contrasting results from the trace-fossil record with models of diversity portioning based on comprehensive body-fossil datasets.

The absence of distinct patterns between trace fossils and particular sedimentary environments reveals the dominance of facies-crossing ichnogenera during the Ediacaran. This pattern is consistent with the observation that most Ediacaran trace-fossil assemblages are exclusively composed of simple grazing trails^3^. This suggests that ecological opportunistic strategies, reflecting low levels of habitat specialization in marine settings, were instrumental in the early colonization of empty or underutilized ecospace in a wide variety of environments^41^. Ediacaran animal-substrate interactions initially developed in an ecological vacuum similar to that of the aftermath of major environmental disturbances. The continuation of these opportunistic styles of animal-sediment interactions in the Phanerozoic is, therefore, unsurprising, representing clear evidence of the persistence of these very simple grazing patterns.

The low levels of habitat specialization revealed by the trace-fossil record may be seen as contrasting with the emerging picture from the analysis of body fossils, which seems to reveal more ecological complexity than initially thought^42^. Recent studies on the Ediacara biota emphasize the development of complex communities consisting of species that competed for different resources occupying a wide range of ecologies^42^. Also, comparative analyses of the Ediacara biota have revealed unusually high patchiness^43,44^. Interestingly, it has been noted in the Flinders Ranges deposits that one of the few forms in the assemblages showing evidence of motility, *Dickinsonia*, occurs on nearly all layers in the facies studied, suggesting that dispersal limitations in sessile organisms may have contributed to patchiness^43^. With the exception of *Kimberichnus* and *Epibaion*, produced by iconic members of the Ediacara biota (regardless of their phylogenetic affinities)^45–48^, the Ediacaran trace-fossil record comprises for the most part Phanerozoic-type ichnotaxa that continued into the Cambrian. Accordingly, they reveal a low level of habitat specialization for the emerging bilaterian communities, although this may not necessarily be the case for the Ediacara biota itself^42,44^. The contrasting pattern in ecological complexity and habitat segregation shown by the Ediacaran body- and trace-fossil record may reflect the different nature of the emerging bilaterian biota, essentially its motility and opportunistic character.

This picture dramatically changed during the early Cambrian, initially with the diversification that took place during the Fortunian and later with the profound ecologic changes during Cambrian Ages 2 to 4^3,4,6,12,19,49^. Overall, the Agronomic Revolution has two major consequences. First, diversification of styles of animal-substrate interactions resulted in the establishment of distinct endobenthic communities along the marine depositional profile in areas landwards of the slope break. This is clearly evidenced by the segregation of two major communities: a deep-tier endobenthic community dominated by sessile predators and suspension feeders in moderate- to high-energy nearshore sands, and a shallow- to intermediate-tier in moderate- to low-energy settings below fair-weather wave base dominated by active predators, detritus feeders and deposit feeders, which correspond to the archetypal *Skolithos* and *Cruziana* ichnofacies, respectively^14^. Second, progressive habitat segregation also took place in response to matground environmental restriction during the Agronomic Revolution. CA shows that *Oldhamia*, the archetypal Cambrian matground-related ichnogenus, experienced a migration from shallow-marine environments into deep-marine settings. The increase of bioturbation in shallow-marine settings that took place during the Agronomic Revolution was detrimental for extensive matground development in nearshore and offshore areas. In this regard, the seaward migration of *Oldhamia* may track in part the restriction of the matground biotope. Accordingly, a third type of community became established along the depositional profile, a relic one of Ediacaran style dominated by the undermat mining ichnogenus *Oldhamia* in deep-marine deposits^50^. Migration of *Oldhamia* provides additional evidence of the impact of bioturbation in shallow-marine settings under fully marine conditions.

The influences of *Bergaueria* in both marginal- and shallow-marine depositional environments may be explained by the fact that its producers, actinians^51^ and cerianthids^52^, may prefer a distinct set of ecological conditions present at these depositional environments. Burrowing anemones, in particular cerianthid anemones, produce relatively deep structures, being able to retract into the burrow by anchoring its expanded physa^53^. Also, cerianthid anemones are able to reposition their burrows in response to vertical accretion of the sea floor^54^. Accordingly, these organisms are well suited to colonize marginal- and shallow-marine settings affected by rapid fluctuations in erosion and sedimentation rates, such as deltas and storm-influenced shallow-marine environments^55^.

CA provides further support to the notion that the Agronomic Revolution was diachronic, progressing from shallow-marine environments into deeper-water ones^12,14^. Colonization of marginal-marine environments during the Cambrian was incipient^27,56–58^, and it is still difficult to quantify based on this methodological approach. Our study also provides further evidence underscoring the extent and significance at the ecosystem scale of changes in bioturbation that took place during Cambrian Ages 2–4 in shallow-marine settings. The fact that an increase in alpha ichnodiversity^3^ took place parallel to an increase in habitat segregation during the early Cambrian is consistent with progressive infaunalization leading to increased ecospace colonization.

There is considerable debate regarding the comparative impact of competition, predation, and physical disturbance in macroevolution^21,59,60^. Habitat segregation during the Cambrian radiation has been explained by assuming a low-competition model, which may have been controlled by niche contraction^21^. Within the scenario of low competition, the addition of new species to a community takes place by exploitation of previously underutilized resources or by packing more species in marginal ecospace. Progressive increase in alpha diversity typically results in increased competition, niche contraction, and increased beta diversity. This is consistent with trace-fossil data showing that both alpha and beta ichnodiversity increased during the early Cambrian, becoming major contributions to global ichnodiversity^3,12^.

The principle of trophic amensalism has been invoked to explain that deposit feeders may negatively affect suspension feeders to the extent of making life impossible for the latter^61^. The segregation of the two early Cambrian platform communities indicated in our analysis may be understood along these lines, as these two are characterized by the dominance of contrasting feeding types. Large, mobile, detritus and deposit feeders that occupied shallow to intermediate tiers in early Cambrian fine-grained offshore muddy sediment may have negatively affected sessile suspension feeders. This effect was most likely produced through sediment reworking that resulted in destabilization of the substrate and resuspension of fine-grained sediment that clogged the filtering structures of suspension feeders. In contrast, deep-tier, mucus-lined vertical domiciles of sessile suspension feeders and predators in nearshore sand tended to decrease resuspension and erosion, stabilizing the sediment. In addition, the concept of trophic amensalism can be expanded to explain other types of habitat segregation, such as the restriction of matground-related trophic types into deeper water. In this case, limited vertical bioturbation in the Cambrian deep sea allowed for the persistence of microbial mats that were host to a variety of a superficial to very shallow-tier benthos that developed sophisticated strategies to feed from these microbial resources^50^.

One of the strengths of the trace-fossil record is its potential to reveal ecological changes at different levels. Also, the intimate link between trace fossils, substrates and depositional environments makes the trace-fossil record particularly useful to frame behavioral innovations in specific environmental settings to a degree which is usually rare to achieve with body fossils. Styles of animal-substrate interactions dramatically changed during the early Cambrian^3^. Although the roots of the Cambrian explosion can be traced to the Ediacaran^32,39,62–66^, analysis of the trace-fossil record shows that early Cambrian bilaterian communities were ecologically distinct and markedly different with respect to their Ediacaran counterparts. Increased habitat segregation during the early Cambrian is consistent with the notion that an increased complexity and heterogeneity of marine ecosystems during the Ediacaran-Cambrian transition may have been a major force of evolutionary change^67,68^.

## Methods

### Paleoenvironmental categories

We have followed previously used depositional environment subdivisions in ichnologic studies^12,69^. To provide a level of consistency among these reported subdivisions, a standardized list was created (see supplementary material Table S1). As part of the comprehensive review of published material, reported depositional environments were categorized and captured at the highest level of detail possible. Given the link between trace fossils and sedimentary facies, ichnologic studies typically benefit from high-resolution paleoenvironmental information to a degree not always reached in body-fossil studies. This fact allows us to produce a finely tuned integration of the sedimentologic and ichnologic datasets to evaluate patterns of distribution of trace fossils.

In this scheme, marginal-marine environments are represented by those settings strongly affected by rapid salinity fluctuations. This category includes bays, estuarine channels and basins, distributary channels, interdistributary bays, lagoons, and mouth bars. Shallow-marine environments encompass all those ranging between the coastal zone to the shelf break. In wave-dominated settings, these include backshore, foreshore, shoreface, offshore, and shelf. In tide-dominated settings, these include the supratidal, intertidal, and subtidal zones. Deep-marine environments comprise those settings located beyond the shelf break, and mostly include turbidite systems present either on the slope or at the base of slope. Subenvironments typically are represented by channels, levees, crevasse splays, and terminal splays.

### Ordination using CA

Ordination is a mathematical technique used to summarize and describe the underlying structure and patterns within multivariate data^36,70,71^. This technique converts a dataset with a high number of dimensions into a dataset with a reduced number of dimensions (ideally two or three) that can then be plotted without undue loss of information^71^. Of the multiple ordination techniques, CA was selected for use as this method can process nominal data^70^.

CA requires the creation of a contingency table between two variables^36^, for this study the contingency table consists of ichnogenera counts observed within different depositional environments. CA uses the contingency table to calculate the chi-squared (χ^2^) distance to quantify the relationships that exist between the rows and columns for every cell, producing eigenvectors and associated eigenvalues^36,71^. Eigenvectors represent the new dimensions produced from the ordination, while the eigenvalues distinguish how much of the variation from the original dataset is described by each eigenvector^71^.

Another important characteristic of the chi-squared distance used in CA, is that it is not influenced by double zeros^40^. This characteristic makes CA particularly suitable for the analysis of species abundance data, as zero values may have several ecological meanings, which make them difficult to interpret. Ecologically, the presence of a species (nonzero value) suggests a site met the minimal conditions for species survival, whereas the absence (zero value) may be caused by a variety of circumstances^40^. Examples include the occupation of an ecological habitat by a replacement species, the species has simply not reached the site despite favorable ecological conditions, the species may not show a regular distribution of the sites studied, the site does not contain favorable ecological conditions for the species, or the species is present but not observed. To summarize, the presence of double zeros cannot be interpreted as resemblance because the absence may be caused by different reasons, including physical (i.e., environmental) and biological (i.e., developmental, ecologic) constraints.

### Data reduction to meet CA requirements

To conduct CA, a contingency table must be created from a dataset in which rows (*r*) represent observations and columns (*c*) represent variables^70^. CA assumes *r* ≥ *c*, a condition that must be accounted for with the creation of a contingency table from the ichnology data. In this study, rows (or ‘observations’) are represented by depositional environments and columns (or ‘variables’) are represented by trace fossils.

Data was compiled, stored, and extracted using IchnoDB^72^, resulting in a dataset that violated the *r* ≥ *c* requirement due to the large number of ichnotaxa present across the time period of interest. To address this problem, ichnotaxa were considered at ichnogenus level instead of at ichnospecies level. This was both practical and appropriate, as taxonomic classification at ichnogenus rank is more thoroughly established than at the ichnospecies level^69^ and this supported the observation of beta ichnodiversity along depositional environments^73–75^. Despite this type of variable grouping, columns still far outnumbered the rows present in contingency tables. To further reduce the number of columns, ichnogenera that contain frequency counts of 1 are removed from the contingency table, followed by progressively higher frequency counts until the number of columns removed satisfies the *r* ≥ *c* criteria for CA.

The ichnology dataset used for CA is subject to bias, specifically ichnotaxonomic classification and interpretation of depositional environments. Potential bias within individual ichnotaxonomic classifications is addressed through the use of ichnogenera rather than ichnospecies. In addition, a comprehensive and critical review of published Ediacaran and Cambrian trace fossils provides a level of consistency in the ichnotaxonomic classifications^12^.

Having defined the data requirements, intervals of time had to be established for analysis. The largest influencing factor in determining intervals was previous trace-fossil research during the ecological transition^3^ from the Ediacaran to early Cambrian. Three intervals were selected, being the Ediacaran, the Terreneuvian, and the Cambrian Series 2. This is roughly coincident with major ecological and evolutionary events. Further, the selection of time intervals at the Series scale promoted larger sample sizes for analysis, as robust multivariate analysis would require the exclusion of trace-fossil records that are not restricted to a defined interval. In addition, resolution at Series level is the most appropriate given available biostratigraphic and chronostratigraphic information^12^.

### Utilizing and understanding biplots

CA analysis produces descriptor-axes (eigenvalues) and object-vectors (eigenvectors) that can be plotted in fewer dimensions than the original data (typically two), which if plotted together result in the formation of a biplot^35,36^. However, it must be noted that CA analysis produces n-1 descriptor-axes than the original number of columns in the dataset^35^. The first axis represents the largest amount of variance within the dataset, with subsequent axes representing decreasing amounts of variance. Descriptor-axes (eigenvalues) are commonly plotted as a bar plot, referred to as a scree plot, to evaluate the total variance explained by each axis^76^. The scree plot is commonly used as a guide to determine how many of the resulting axes should be retained for analysis. Two common methods are: identifying the inflection point in the plot and only keeping axes above this cut-off^77^, and determining the average expected variation for each column to use as the guide for axis retention^34,76^.

Biplots reveal structure in the data that may not have otherwise been evident by displaying patterns of correlations between variables or relationships between observations^76^. As a general guide to the interpretation of biplots, variables (i.e., ichnogenera) with large contributions to the position of the objects (i.e., depositional environments) will be close to the object of interest on the plot^35^. Variables that are found in close proximity to an object are likely to be present or in greater abundance than variables further away^36^.

The interpretation of biplots should not focus on the linear distance between variables and objects, rather it is the angle between points that is significant in uncovering relationships^34^. Drawing a line between a point on the biplot and the origin can help in more effectively determining the angle between points, where an acute angle indicates a positive association, a right angle indicates no association, and an obtuse angle indicates a negative association. The contribution and quality of representation for the variables and objects are additional metrics that are helpful in biplot interpretation. Contribution is the extent to which a data point contributes to a dimension^78^, highlighting the most significant variables and objects for any given biplot. Quality of representation explains how well a variable or object is summarized in the two newly created dimensions of the ordination, with a value close to 1 indicating points that are well represented and a value close to 0 indicating points that are poorly represented.^34,78^. Any value over 0.66 was classified as well represented, 0.33 to 0.66 was classified as moderately represented, and values less than 0.33 were classified as poorly represented. These metrics are particularly helpful, as the chi-square distance used in CA is particularly susceptible to variables with small frequency counts, which plot on the periphery of the biplot and often lead to the incorrect interpretation of a highly significant occurrence^36^.

The FactoMineR package for R^33^ was used to conduct CA and produce figures. Although other packages in R^79^ support CA (e.g., vegan^80^, ca^81^), FactoMineR was easy to use and the supplementary factoextra package^82^ supported figure generation. The scripted code used to import the data and create figures for interpretation is included in the supplementary materials for the sake of reproducibility.

### Supplementary Information

Supplementary Information 1.

Supplementary Information 2.

## Data Availability

All data generated or analyzed during this study are included in this published article (and its Supplementary Information files).
