# Supplementary material for: Increased habitat segregation at the dawn of the Phanerozoic revealed by correspondence analysis of bioturbation
Source: Sci Rep. 2023 Dec 15;13:22328. doi: 10.1038/s41598-023-49716-8 (PMC10724277; doi:10.1038/s41598-023-49716-8)
Supplement: Supplementary file 1 — Supplementary Information 1. [file 41598_2023_49716_MOESM1_ESM.docx]

Supplementary Information for

**Increased habitat segregation at the dawn of the Phanerozoic revealed by correspondence analysis of bioturbation**

Dean M. Meek^1^, Luis A. Buatois^1^, M. Gabriela Mángano^1^, Bruce M. Eglington^1^

^1^Department of Geological Sciences, University of Saskatchewan, Saskatoon, SK S7N 5E2, Canada. *Correspondence and requests for materials should be addressed to D.M.M. (email: d.meek@usask.ca)

**This PDF file includes:**

Tables S1 to S4

Figures S1 to S30

R Code

**Table S1**

Depositional environments identified to provide standardization for data compilation. Classification is provided to the highest level where possible (e.g., shallow marine wave dominated offshore; SHMWDOF), while simplification to the “parent” depositional environment is necessary where uncertainties exist (e.g., shallow marine wave dominated; SHMWD). Therefore, classification is not necessarily mutually exclusive.

| **Dep. Env. Abr.** | **Description of Depositional Environment** | **Dep. Env. Parent Abr.** |
| --- | --- | --- |
| 0 | Not Defined | - |
| CNT | Continental | - |
| CNTE | Continental - eolian | CNT |
| CNTED | Eolian - dune | CNTE |
| CNTEID | Eolian - interdune | CNTE |
| CNTESS | Eolian - sandsheets | CNTE |
| CNTF | Continental - fluvial | CNT |
| CNTFA | Fluvial - anastomosing | CNTF |
| CNTFAC | Anastomosing - channel | CNTFA |
| CNTFAOV | Anastomosing - overbank | CNTFA |
| CNTFB | Fluvial - braided | CNTF |
| CNTFBC | Braided - channel | CNTFB |
| CNTFBOV | Braided - overbank | CNTFB |
| CNTFE | Fluvial - ephemeral | CNTF |
| CNTFM | Fluvial - meandering | CNTF |
| CNTFMC | Meandering - channel | CNTFM |
| CNTFMOV | Meandering - overbank | CNTFM |
| CNTL | Continental - lake | CNT |
| CNTLM | Lake - lake margin | CNTL |
| CNTLSAQ | Lake - subaqueous | CNTL |
| DM | Deep Marine | - |
| DMB | Deep marine - basin | DM |
| DMS | Deep marine - slope | DM |
| DMTS | Deep Marine - turbidite system | DM |
| DMTSC | Turbidite system - channel | DMTS |
| DMTSL | Turbidite system - levee | DMTS |
| DMTSSC | Turbidite system - crevasse splay | DMTS |
| DMTSST | Turbidite system - terminal splay | DMTS |
| MM | Marginal marine | - |
| MMD | Marginal marine - delta | MM |
| MMDRD | Delta - river dominated | MMD |
| MMDRDDF | River dominated - delta front | MMDRD |
| MMDRDDP | River dominated - delta plain | MMDRD |
| MMDRDPD | River dominated - prodelta | MMDRD |

**Table S1 Continued.**

| **Dep. Env. Abr.** | **Description of Depositional Environment** | **Dep. Env. Parent Abr.** |
| --- | --- | --- |
| MMDTD | Delta - tide dominated | MMD |
| MMDTDDF | Tide dominated - delta front | MMDTD |
| MMDTDDP | Tide dominated - delta plain | MMDTD |
| MMDTDPD | Tide dominated - prodelta | MMDTD |
| MMDWD | Delta - wave dominated | MMD |
| MMDWDDF | Wave dominated - delta front | MMDWD |
| MMDWDDP | Wave dominated - delta plain | MMDWD |
| MMDWDPD | Wave dominated - prodelta | MMDWD |
| MME | Marginal marine - estuary | MM |
| MMETD | Estuary - tide dominated | MME |
| MMETDIEC | Tide dominated - inner estuary channels | MMETD |
| MMETDMEC | Tide dominated - middle estuary channels | MMETD |
| MMETDSSTF | Tide dominated - subtidal sandbars / tidal flats | MMETD |
| MMEWD | Estuary - wave dominated | MME |
| MMEWDBD | Wave dominated - bayhead delta | MMEWD |
| MMEWDEB | Wave dominated - estuary basin | MMEWD |
| MMEWDEM | Wave dominated - estuary mouth | MMEWD |
| SHM | Shallow marine | - |
| SHMPT | Shallow marine - platform | SHM |
| SHMPTI | Platform - inner | SHMPT |
| SHMPTM | Platform - middle | SHMPT |
| SHMPTO | Platform - outer | SHMPT |
| SHMPTopn | Shallow marine - open platform | SHMPT |
| SHMPTrstd | Shallow marine - restricted platform | SHMPT |
| SHMRF | Shallow marine - reef | SHM |
| SHMRP | Shallow marine - ramp | SHM |
| SHMRPI | Ramp - inner | SHMRP |
| SHMRPM | Ramp - middle | SHMRP |
| SHMRPO | Ramp - outer | SHMRP |
| SHMTD | Shallow marine - tide dominated | SHM |
| SHMTDI | Tide dominated - intertidal | SHMTD |
| SHMTDITC | Intertidal - tidal channels | SHMTDI |
| SHMTDITF | Intertidal - tidal flat | SHMTDI |
| SHMTDITFFM | Tidal flat - upper mud flat | SHMTDITF |
| SHMTDITFFS | Tidal flat - lower sand flat | SHMTDITF |
| SHMTDITFFX | Tidal flat - middle mixed flat | SHMTDITF |
| SHMTDSBT | Tide dominated - subtidal | SHMTD |
| SHMTDSPT | Tide dominated - supratidal | SHMTD |
| SHMWD | Shallow marine - wave dominated | SHM |
| SHMWDBS | Wave dominated - backshore | SHMWD |

**Table S1 Continued.**

| **Dep. Env. Abr.** | **Description of Depositional Environment** | **Dep. Env. Parent Abr.** |
| --- | --- | --- |
| SHMWDFS | Wave dominated - foreshore | SHMWD |
| SHMWDOF | Wave dominated - offshore | SHMWD |
| SHMWDOFL | Offshore - lower offshore | SHMWDOF |
| SHMWDOFT | Offshore - transition | SHMWDOF |
| SHMWDOFU | Offshore - upper offshore | SHMWDOF |
| SHMWDSF | Wave dominated - shoreface | SHMWD |
| SHMWDSFL | Shoreface - lower shoreface | SHMWDSF |
| SHMWDSFM | Shoreface - middle shoreface | SHMWDSF |
| SHMWDSFU | Shoreface - upper shoreface | SHMWDSF |
| SHMWDSH | Wave dominated - shelf | SHMWD |

**Table S2**

Contingency table created from compiled trace fossil occurrences during the Ediacaran. Individual cells are shaded to aid in the identification of high frequency occurrences in relation to all other values in the table. Both column summation (bottom row) and row summation (rightmost column) are included, and display individual greyscale shading to aid in the identification of larger frequency count summations in relation to other column or row summations. Abbreviations are as follows: *Archaeonassa* (*An*), *Bergaueria* (*Be*), *Gordia* (*Go*), *Helminthoidichnites* (*He*), *Helminthopsis* (*Hl*), *Kimberichnus* (*Km*), *Palaeophycus* (*Pa*), *Torrowangea* (*To*), *Treptichnus* (*Tr*); Deep marine (DM), Deep marine turbidite system (DMTS), Marginal marine deltaic (MMD), Marginal marine deltaic – wave dominated (MMDWD), Marginal marine deltaic – wave dominated delta front (MMDWDDF), Shallow marine (SHM), Shallow marine tide dominated (SHMTD), Shallow marine tide dominated – Intertidal (SHMTDI), Shallow marine tide dominated – supratidal (SHMTDSPT), Shallow marine wave dominated (SHMWD), Shallow marine wave dominated – offshore (SHMWDOF), Shallow marine wave dominated – offshore transition (SHMWDOFT), Shallow marine wave dominated – offshore transition (SHMWDOFT), Shallow marine wave dominated – shelf (SHMWDSH).

|  | ***An*** | ***Be*** | ***Go*** | ***He*** | ***Hl*** | ***Km*** | ***Pa*** | ***To*** | ***Tr*** |  |
| --- | --- | --- | --- | --- | --- | --- | --- | --- | --- | --- |
| **DM** | 1 | 0 | 0 | 11 | 8 | 0 | 10 | 5 | 1 | *36* |
| **DMTS** | 0 | 0 | 4 | 0 | 0 | 0 | 2 | 1 | 0 | *7* |
| **MMD** | 3 | 0 | 0 | 0 | 0 | 0 | 0 | 0 | 0 | *3* |
| **MMDWD** | 0 | 0 | 2 | 0 | 0 | 0 | 2 | 0 | 0 | *4* |
| **MMDWDDF** | 0 | 0 | 3 | 0 | 0 | 0 | 0 | 0 | 0 | *3* |
| **SHM** | 1 | 0 | 0 | 3 | 1 | 0 | 7 | 0 | 0 | *12* |
| **SHMTD** | 0 | 0 | 4 | 1 | 1 | 0 | 8 | 3 | 2 | *19* |
| **SHMTDI** | 0 | 0 | 0 | 0 | 0 | 0 | 2 | 1 | 0 | *3* |
| **SHMTDSBT** | 0 | 0 | 0 | 1 | 0 | 0 | 1 | 0 | 0 | *2* |
| **SHMTDSPT** | 0 | 1 | 1 | 0 | 1 | 0 | 2 | 2 | 0 | *7* |
| **SHMWD** | 0 | 0 | 0 | 0 | 0 | 0 | 0 | 0 | 2 | *2* |
| **SHMWDOF** | 3 | 4 | 0 | 9 | 2 | 5 | 8 | 1 | 0 | *32* |
| **SHMWDOFT** | 2 | 0 | 1 | 1 | 0 | 1 | 1 | 0 | 0 | *6* |
| **SHMWDOFU** | 0 | 0 | 0 | 1 | 0 | 0 | 0 | 0 | 0 | *1* |
| **SHMWDSH** | 0 | 0 | 1 | 0 | 0 | 0 | 5 | 0 | 0 | *6* |
|  | *10* | *5* | *16* | *27* | *13* | *6* | *48* | *13* | *5* |  |

**Table S3**

Contingency table created from compiled trace fossil occurrences during the Terreneuvian. Individual cells are shaded to aid in the identification of high frequency occurrences in relation to all other values in the table. Both column summation (bottom row) and row summation (rightmost column) are included, and display individual greyscale shading to aid in the identification of larger frequency count summations in relation to other column or row summations. Abbreviations are as follows: *Arenicolites* (*Ar*), *Cochlichnus* (*Co*), *Cruziana* (*Cu*), *Didymaulichnus* (*Dd*), *Diplocraterion* (*Dp*), *Gordia* (*Go*), *Gyrolithes* (*Gy*), *Helminthoidichnites* (*He*), *Helminthopsis* (*Hl*), *Monomorphichnus* (*Mo*), *Oldhamia* (*Ol*), *Palaeophycus* (*Pa*), *Planolites* (*Pl*), *Psammichnites* (*Ps*), *Rusophycus* (*Ru*), *Skolithos* (*Sk*), *Treptichnus* (*Tr*); Deep marine turbidite system (DMTS), Marginal marine (MM), Marginal marine estuary (MME), Marginal marine deltaic (MMD), Shallow marine (SHM), Shallow marine platform (SHMPT), Shallow marine tide dominated (SHMTD), Shallow marine tide dominated – intertidal (SHMTDI), Shallow marine tide dominated – intertidal tidal flat (SHMTDITF), Shallow marine tide dominated – subtidal (SHMTDSBT), Shallow marine wave dominated (SHMWD), Shallow marine wave dominated – shoreface (SHMWDSF), Shallow marine wave dominated – shoreface lower (SHMWDSFL), Shallow marine wave dominated – offshore (SHMWDOF), Shallow marine wave dominated – offshore lower (SHMWDOFL), Shallow marine wave dominated – offshore transition (SHMWDOFT), Shallow marine wave dominated – offshore upper (SHMWDOFU), Shallow marine wave dominated – shelf (SHMWDSH).

|  | ***Ar*** | ***Co*** | ***Cr*** | ***Dd*** | ***Dp*** | ***Go*** | ***Gy*** | ***He*** | ***Hl*** | ***Mo*** | ***Ol*** | ***Pa*** | ***Pl*** | ***Ps*** | ***Ru*** | ***Sk*** | ***Tr*** |  |
| --- | --- | --- | --- | --- | --- | --- | --- | --- | --- | --- | --- | --- | --- | --- | --- | --- | --- | --- |
| **DMTS** | 0 | 2 | 0 | 2 | 0 | 0 | 0 | 2 | 4 | 0 | 11 | 2 | 0 | 0 | 0 | 0 | 0 | 23 |
| **MM** | 0 | 0 | 0 | 0 | 0 | 0 | 0 | 1 | 0 | 0 | 0 | 5 | 4 | 1 | 0 | 0 | 0 | 11 |
| **MME** | 0 | 1 | 0 | 0 | 0 | 0 | 0 | 0 | 0 | 0 | 0 | 0 | 1 | 0 | 0 | 1 | 0 | 3 |
| **MMD** | 0 | 0 | 0 | 1 | 4 | 0 | 0 | 0 | 0 | 0 | 0 | 2 | 1 | 0 | 2 | 2 | 0 | 16 |
| **SHM** | 1 | 2 | 0 | 4 | 1 | 0 | 2 | 0 | 0 | 4 | 0 | 4 | 3 | 1 | 1 | 2 | 11 | 36 |
| **SHMPT** | 0 | 0 | 0 | 0 | 1 | 0 | 0 | 0 | 0 | 0 | 0 | 0 | 2 | 0 | 0 | 0 | 0 | 3 |
| **SHMTD** | 1 | 0 | 1 | 1 | 2 | 0 | 0 | 0 | 0 | 2 | 0 | 1 | 3 | 1 | 0 | 1 | 0 | 13 |
| **SHMTDI** | 1 | 0 | 1 | 1 | 0 | 1 | 0 | 0 | 0 | 0 | 0 | 1 | 1 | 1 | 1 | 0 | 3 | 11 |
| **SHMTDITF** | 2 | 0 | 0 | 0 | 1 | 2 | 0 | 0 | 0 | 0 | 0 | 2 | 0 | 0 | 0 | 3 | 7 | 17 |
| **SHMTDSBT** | 3 | 1 | 4 | 2 | 0 | 3 | 0 | 0 | 0 | 3 | 0 | 7 | 3 | 2 | 3 | 13 | 2 | 46 |
| **SHMWD** | 0 | 0 | 0 | 0 | 0 | 0 | 1 | 0 | 0 | 0 | 0 | 0 | 3 | 0 | 0 | 5 | 4 | 13 |
| **SHMWDSF** | 0 | 0 | 0 | 1 | 1 | 0 | 0 | 0 | 1 | 0 | 0 | 2 | 3 | 1 | 0 | 0 | 1 | 10 |
| **SHMWDSFL** | 1 | 0 | 0 | 0 | 1 | 0 | 0 | 0 | 0 | 0 | 0 | 0 | 2 | 0 | 0 | 1 | 1 | 6 |
| **SHMWDOF** | 3 | 16 | 3 | 7 | 2 | 5 | 6 | 6 | 13 | 17 | 9 | 8 | 25 | 11 | 6 | 5 | 26 | 168 |
| **SHMWDOFL** | 0 | 1 | 1 | 0 | 0 | 1 | 1 | 2 | 1 | 0 | 0 | 2 | 1 | 0 | 0 | 0 | 3 | 13 |
| **SHMWDOFT** | 3 | 2 | 3 | 6 | 3 | 2 | 1 | 1 | 1 | 5 | 0 | 4 | 9 | 3 | 4 | 13 | 9 | 69 |
| **SHMWDOFU** | 0 | 0 | 0 | 0 | 0 | 0 | 0 | 0 | 0 | 0 | 0 | 0 | 0 | 0 | 0 | 0 | 2 | 2 |
| **SHMWDSH** | 3 | 2 | 1 | 1 | 3 | 2 | 3 | 3 | 4 | 2 | 1 | 6 | 21 | 2 | 0 | 2 | 5 | 61 |
|  | 18 | 27 | 14 | 26 | 19 | 16 | 18 | 15 | 24 | 33 | 21 | 46 | 82 | 23 | 17 | 48 | 74 |  |

**Table S4**

Contingency table created from compiled trace fossil occurrences during the Cambrian Epoch 2. Individual cells are shaded to aid in the identification of high frequency occurrences in relation to all other values in the table. Both column summation (bottom row) and row summation (rightmost column) are included, and display individual greyscale shading to aid in the identification of larger frequency count summations in relation to other column or row summations. Abbreviations are as follows: *Bergaueria* (*Be*), *Cruziana* (*Cr*), *Dimorphichnus* (*Dm*), *Diplichnites* (*Di*), *Monomorphichnus* (*Mo*), *Oldhamia* (*Ol*), *Palaeophycus* (*Pa*), *Phycodes* (*Ph*), *Planolites* (*Pl*), *Psammichnites* (*Ps*), *Rosselia* (*Ro*), *Rusophycus* (*Ru*), *Skolithos* (*Sk*), *Teichichnus* (*Te*), *Treptichnus* (*Tr*); Deep marine – turbidite system (DMTS), Marginal marine (MM), Marginal marine deltaic (MMD), Marginal marine deltaic – tide dominated (MMDTD), Shallow marine (SHM), Shallow marine platform (SHMPT), Shallow marine tide dominated (SHMTD), Shallow marine tide dominated – intertidal (SHMTDI), Shallow marine tide dominated – intertidal tidal flat (SHMTDITF), Shallow marine tide dominated – subtidal (SHMTDSBT), Shallow marine wave dominated (SHMWD), Shallow marine wave dominated – foreshore (SHMWDFS), Shallow marine wave dominated – shoreface (SHMWDSF), Shallow marine wave dominated – shoreface lower (SHMWDSFL), Shallow marine wave dominated – offshore transition (SHMWDOFT), Shallow marine wave dominated – offshore (SHMWDOF), Shallow marine wave dominated – shelf (SHMWDSH).

|  | ***Be*** | ***Cr*** | ***Dm*** | ***Di*** | ***Mo*** | ***Ol*** | ***Pa*** | ***Ph*** | ***Pl*** | ***Ps*** | ***Ro*** | ***Ru*** | ***Sk*** | ***Te*** | ***Tr*** |  |
| --- | --- | --- | --- | --- | --- | --- | --- | --- | --- | --- | --- | --- | --- | --- | --- | --- |
| **DMTS** | 1 | 0 | 0 | 0 | 1 | 63 | 45 | 0 | 0 | 3 | 0 | 0 | 0 | 0 | 0 | 113 |
| **MM** | 0 | 1 | 1 | 0 | 2 | 0 | 21 | 3 | 3 | 7 | 0 | 4 | 12 | 0 | 4 | 58 |
| **MMD** | 0 | 0 | 0 | 0 | 0 | 0 | 1 | 0 | 2 | 0 | 0 | 0 | 1 | 2 | 0 | 6 |
| **MMDTD** | 24 | 8 | 0 | 0 | 0 | 0 | 0 | 0 | 8 | 0 | 0 | 8 | 8 | 0 | 0 | 56 |
| **SHM** | 3 | 7 | 7 | 3 | 9 | 0 | 16 | 4 | 15 | 10 | 2 | 11 | 20 | 2 | 7 | 116 |
| **SHMPT** | 1 | 0 | 3 | 2 | 0 | 0 | 3 | 0 | 4 | 3 | 0 | 1 | 1 | 0 | 1 | 19 |
| **SHMTD** | 1 | 2 | 1 | 1 | 2 | 0 | 1 | 3 | 4 | 1 | 2 | 5 | 3 | 5 | 2 | 33 |
| **SHMTDI** | 0 | 0 | 7 | 7 | 0 | 0 | 0 | 0 | 0 | 0 | 0 | 7 | 7 | 0 | 0 | 28 |
| **SHMTDITF** | 0 | 1 | 0 | 0 | 1 | 0 | 1 | 0 | 0 | 0 | 0 | 1 | 2 | 0 | 0 | 6 |
| **SHMTDSBT** | 16 | 31 | 15 | 7 | 4 | 0 | 34 | 25 | 43 | 1 | 37 | 33 | 41 | 21 | 2 | 310 |
| **SHMWD** | 1 | 6 | 1 | 6 | 4 | 0 | 17 | 2 | 14 | 3 | 0 | 1 | 12 | 4 | 8 | 79 |
| **SHMWDFS** | 0 | 0 | 0 | 0 | 0 | 0 | 0 | 0 | 0 | 0 | 0 | 1 | 3 | 0 | 0 | 4 |
| **SHMWDSF** | 4 | 5 | 1 | 3 | 4 | 0 | 12 | 5 | 20 | 5 | 0 | 8 | 13 | 0 | 5 | 85 |
| **SHMWDSFL** | 0 | 0 | 0 | 0 | 0 | 0 | 0 | 0 | 3 | 0 | 0 | 0 | 1 | 1 | 0 | 5 |
| **SHMWDOFT** | 9 | 21 | 5 | 14 | 7 | 0 | 19 | 10 | 45 | 6 | 3 | 36 | 9 | 13 | 8 | 205 |
| **SHMWDOF** | 1 | 9 | 4 | 2 | 9 | 0 | 8 | 1 | 16 | 1 | 0 | 8 | 3 | 0 | 1 | 63 |
| **SHMWDSH** | 4 | 0 | 0 | 0 | 0 | 0 | 1 | 1 | 0 | 0 | 0 | 0 | 2 | 0 | 0 | 8 |
|  | 65 | 91 | 45 | 45 | 43 | 63 | 179 | 54 | 177 | 40 | 44 | 124 | 138 | 48 | 38 |  |

**Fig. S1**

Eigenvalue scree plot for the Ediacaran correspondence analysis. The eigenvalue for each component is plotted with the percentage of representative variance. Included in this figure is the Kaiser-Guttman criterion^1^, recommending the retention of components 1 to 3.


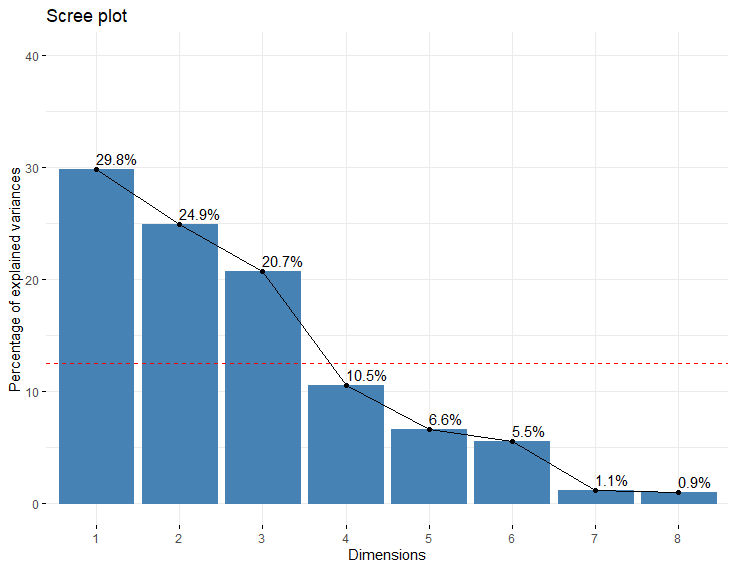


1 Jackson, D. A. Stopping Rules in Principal Components Analysis: A Comparison of Heuristical and Statistical Approaches. *Ecology* **74**, 2204-2214 (1993).

**Fig. S2**

Column (i.e., variable) contribution to the ordination of dimension 1 for the Ediacaran. Red dashed line represents average expected value if data were random. Plot created in R with the factoextra package. Abbreviations provided in table S2 description.


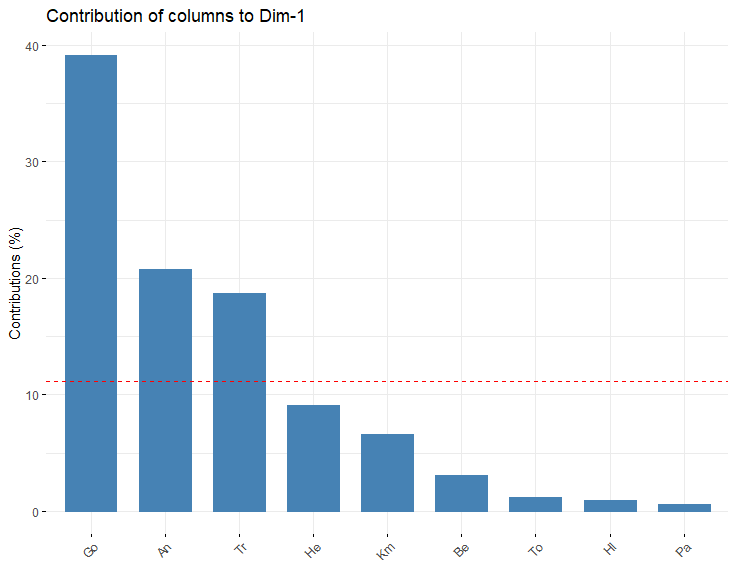


**Fig. S3**

Column (i.e., variable) contribution to the ordination of dimension 2 for the Ediacaran. Red dashed line represents average expected value if data were random. Plot created in R with the factoextra package. Abbreviations provided in table S2 description.


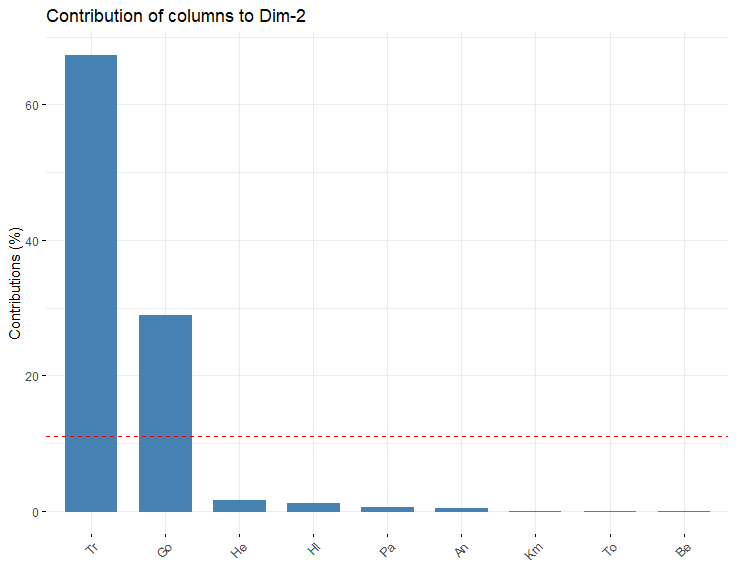


**Fig. S4**

Row (i.e., observation) contribution to the ordination of dimension 1 for the Ediacaran. Red dashed line represents average expected value if data were random. Plot created in R with the factoextra package. Abbreviations provided in table S2 description.


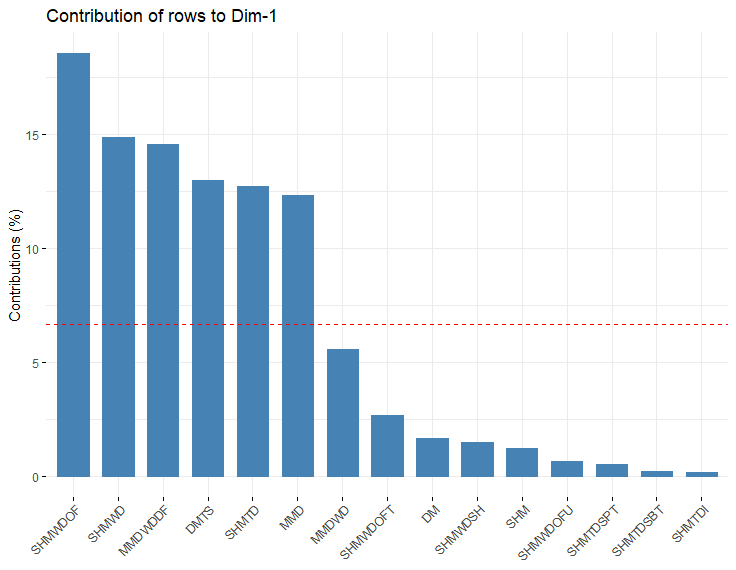


**Fig. S5**

Row (i.e., observation) contribution to the ordination of dimension 2 for the Ediacaran. Red dashed line represents average expected value if data were random. Plot created in R with the factoextra package. Abbreviations provided in table S2 description.


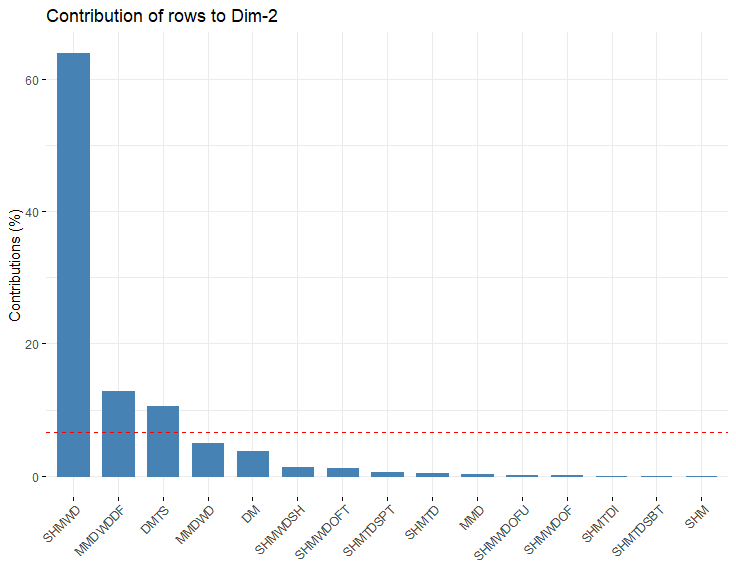


**Fig. S6**

Column (i.e., variable) quality of representation (cos2) for dimensions 1 and 2 of the Ediacaran ordination. Values of ≥0.66 are considered well represented, values ≥0.33 are considered moderately represented, and values <0.33 are considered poorly represented. Plot created in R with the factoextra package. Abbreviations provided in table S2 description.


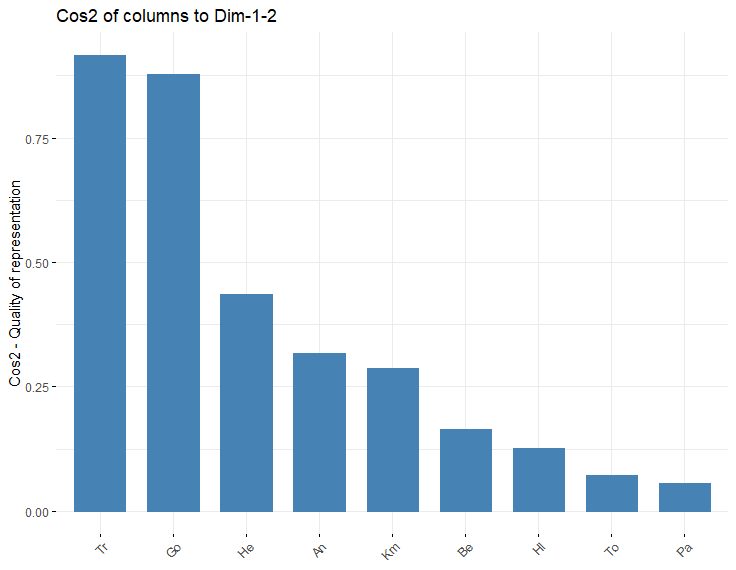


**Fig. S7**

Column (i.e., variable) quality of representation (cos2) for dimensions 1 and 3 of the Ediacaran ordination. Values of ≥0.66 are considered well represented, values ≥0.33 are considered moderately represented, and values <0.33 are considered poorly represented. Plot created in R with the factoextra package. Abbreviations provided in table S2 description.


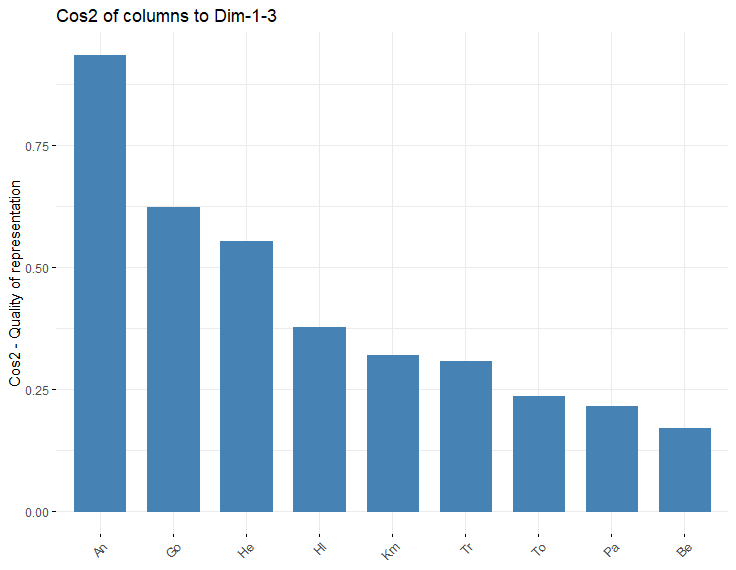


**Fig. S8**

Row (i.e., observation) quality of representation (cos2) for dimensions 1 and 2 of the Ediacaran ordination. Values of ≥0.66 are considered well represented, values ≥0.33 are considered moderately represented, and values <0.33 are considered poorly represented. Plot created in R with the factoextra package. Abbreviations provided in table S2 description.


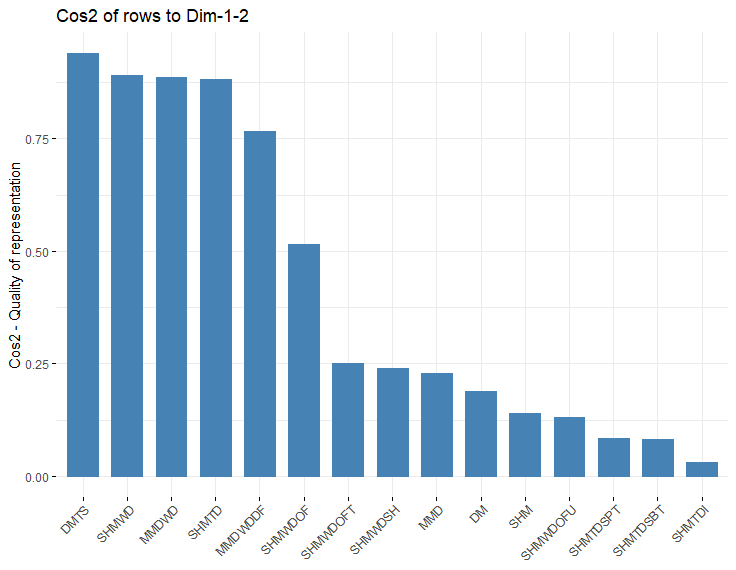


**Fig. S9**

Row (i.e., observation) quality of representation (cos2) for dimensions 1 and 3 of the Ediacaran ordination. Values of ≥0.66 are considered well represented, values ≥0.33 are considered moderately represented, and values <0.33 are considered poorly represented. Plot created in R with the factoextra package. Abbreviations provided in table S2 description.


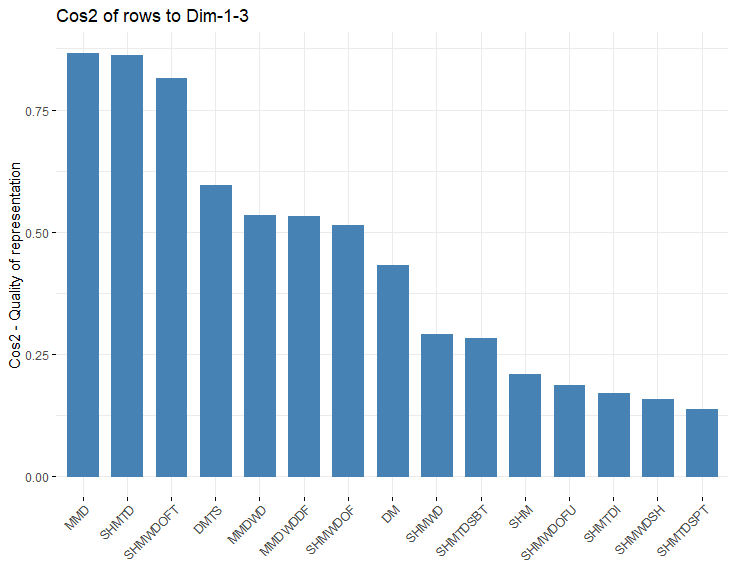


**Fig. S10**

Ediacaran biplot of Components 1 and 3. Plot created in R with the factoextra package. Abbreviations provided in table S2 description.
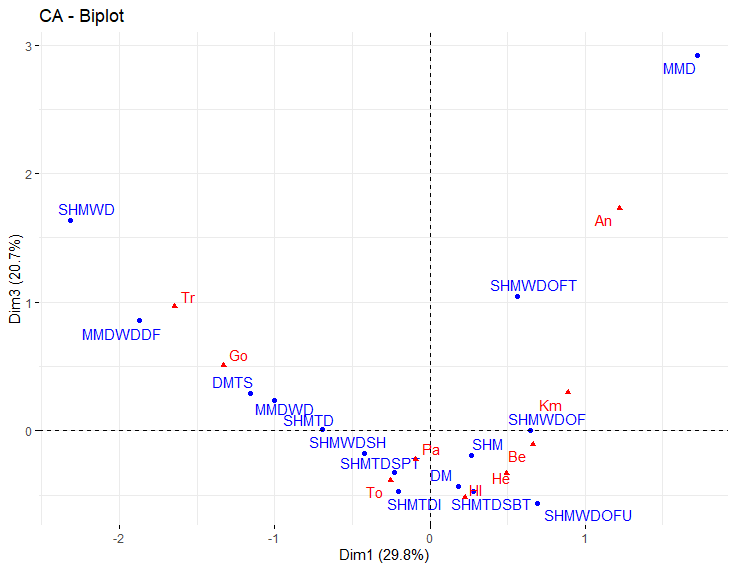


**Fig. S11**

Eigenvalue scree plot for the Terreneuvian correspondence analysis. The eigenvalue for each component is plotted with the percentage of representative variance. Included in this figure is the Kaiser-Guttman criterion, recommending the retention of components 1 to 6; however, only components 1 to 4 were retained for analysis based on the lack of readily explainable patterns in components beyond 1.


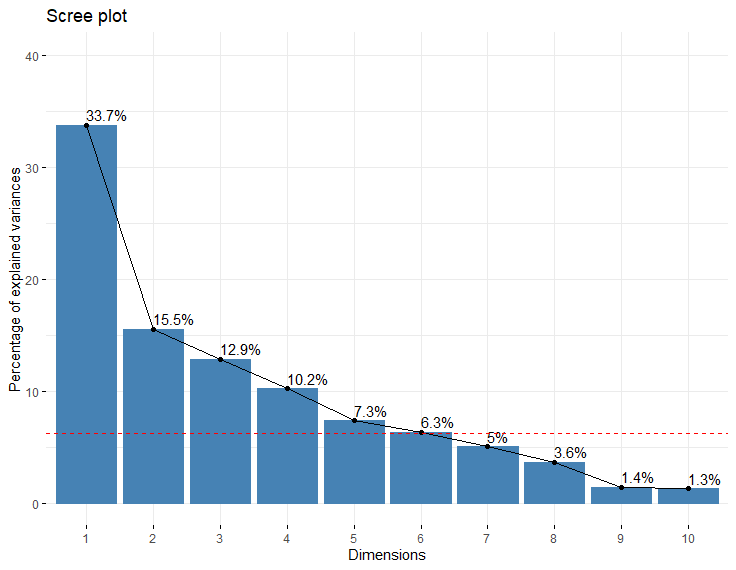


**Fig. S12**

Column (i.e., variable) contribution to the ordination of dimension 1 for the Terreneuvian. Red dashed line represents average expected value if data were random. Plot created in R with the factoextra package. Abbreviations provided in table S3 description.


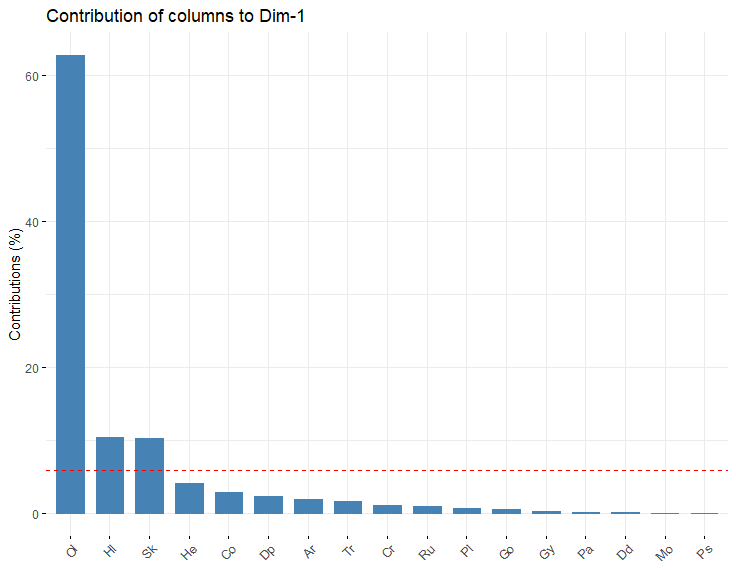


**Fig. S13**

Column (i.e., variable) contribution to the ordination of dimension 2 for the Terreneuvian. Red dashed line represents average expected value if data were random. Plot created in R with the factoextra package. Abbreviations provided in table S3 description.


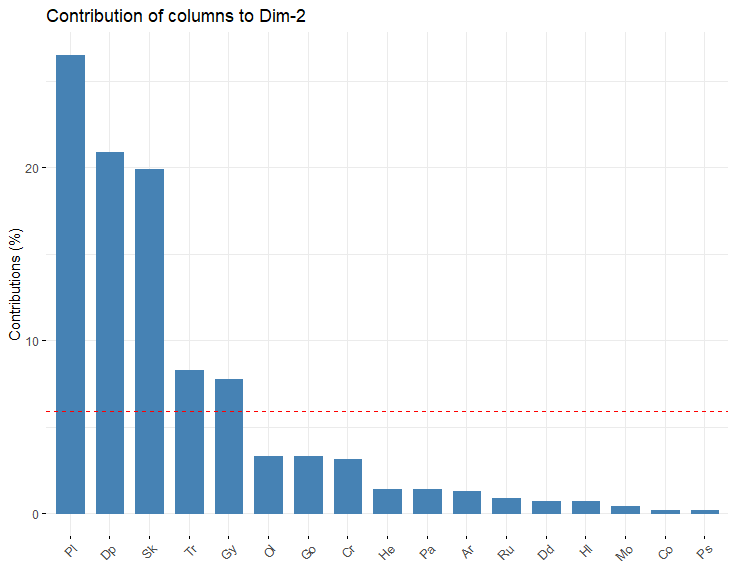


**Fig. S14**

Row (i.e., observation) contribution to the ordination of dimension 1 for the Terreneuvian. Red dashed line represents average expected value if data were random. Plot created in R with the factoextra package. Abbreviations provided in table S3 description.


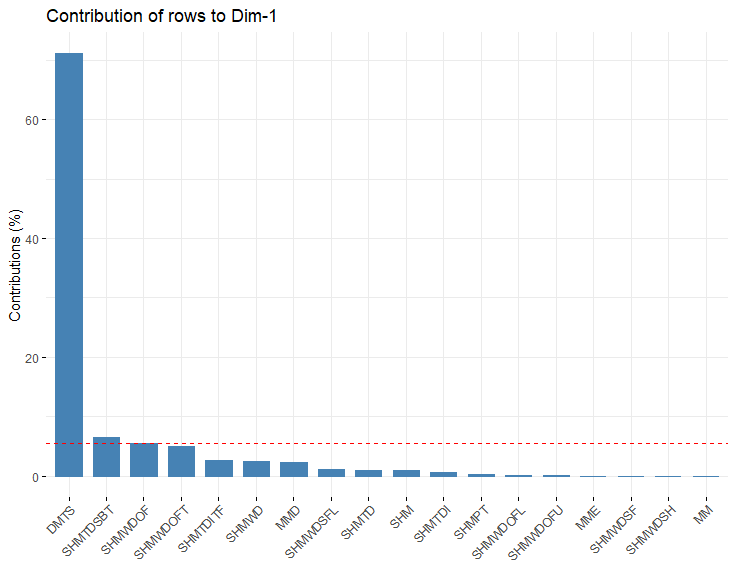


**Fig. S15**

Row (i.e., observation) contribution to the ordination of dimension 2 for the Terreneuvian. Red dashed line represents average expected value if data were random. Plot created in R with the factoextra package. Abbreviations provided in table S3 description.


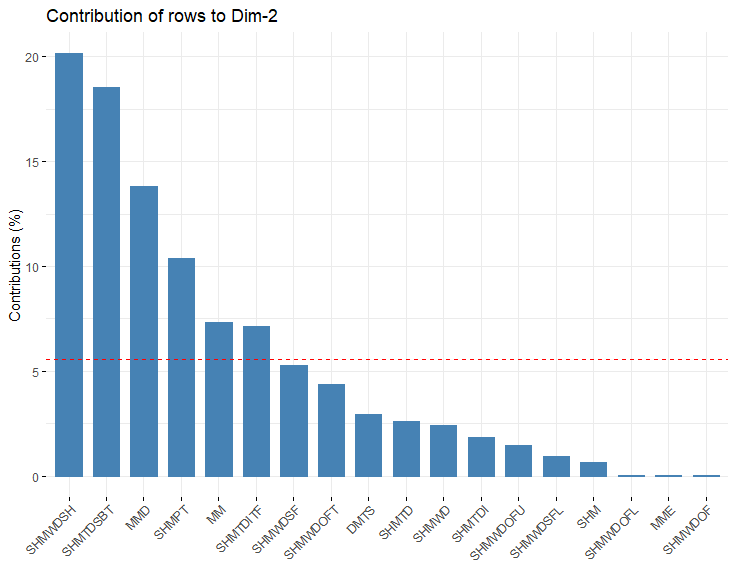


**Fig. S16**

Column (i.e., variable) quality of representation (cos2) for dimensions 1 and 2 of the Terreneuvian ordination. Values of ≥0.66 are considered well represented, values ≥0.33 are considered moderately represented, and values <0.33 are considered poorly represented. Plot created in R with the factoextra package. Abbreviations provided in table S3 description.


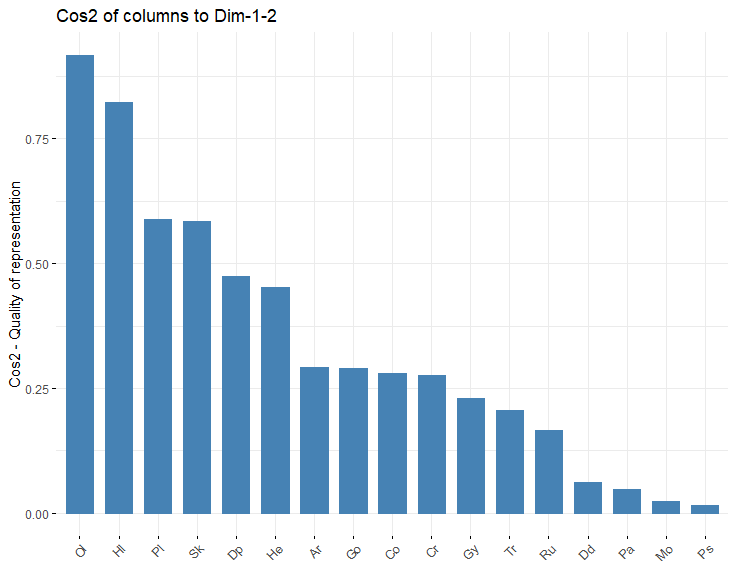


**Fig. S17**

Column (i.e., variable) quality of representation (cos2) for dimensions 3 and 4 of the Terreneuvian ordination. Values of ≥0.66 are considered well represented, values ≥0.33 are considered moderately represented, and values <0.33 are considered poorly represented. Plot created in R with the factoextra package. Abbreviations provided in table S3 description.


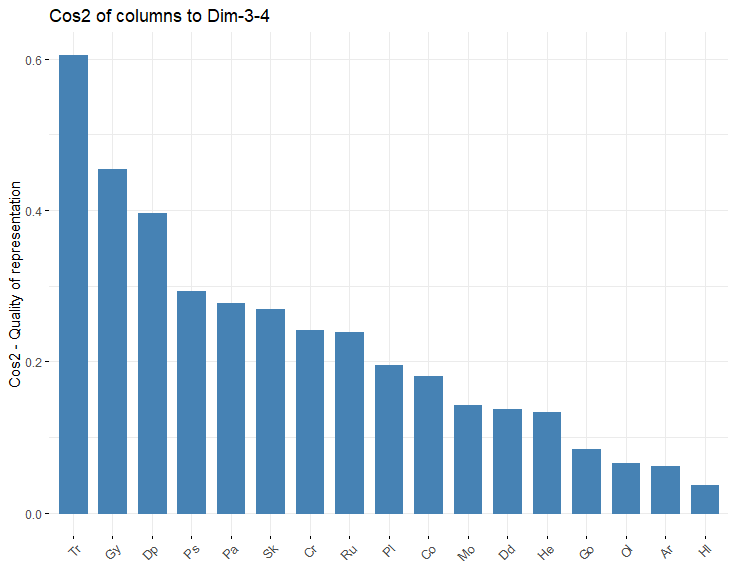


**Fig. S18**

Row (i.e., observation) quality of representation (cos2) for dimensions 1 and 2 of the Terreneuvian ordination. Values of ≥0.66 are considered well represented, values ≥0.33 are considered moderately represented, and values <0.33 are considered poorly represented. Plot created in R with the factoextra package. Abbreviations provided in table S3 description.


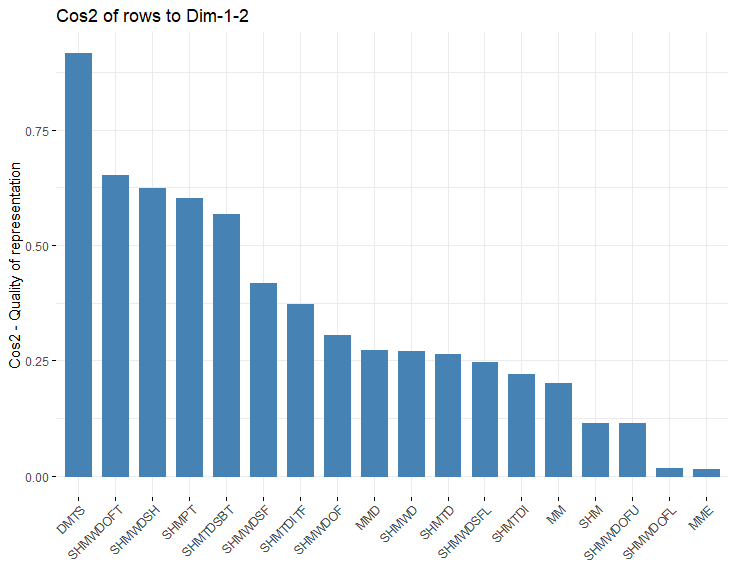


**Fig. S19**

Row (i.e., observation) quality of representation (cos2) for dimensions 3 and 4 of the Terreneuvian ordination. Values of ≥0.66 are considered well represented, values ≥0.33 are considered moderately represented, and values <0.33 are considered poorly represented. Plot created in R with the factoextra package. Abbreviations provided in table S3 description.


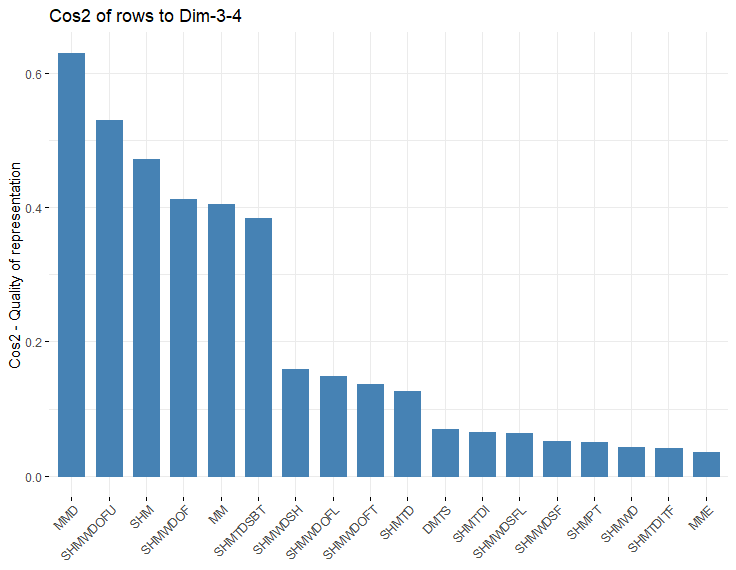


**Fig. S20**

Terreneuvian biplot of Components 3 and 4. Plot created in R with the factoextra package. Abbreviations provided in table S3 description.


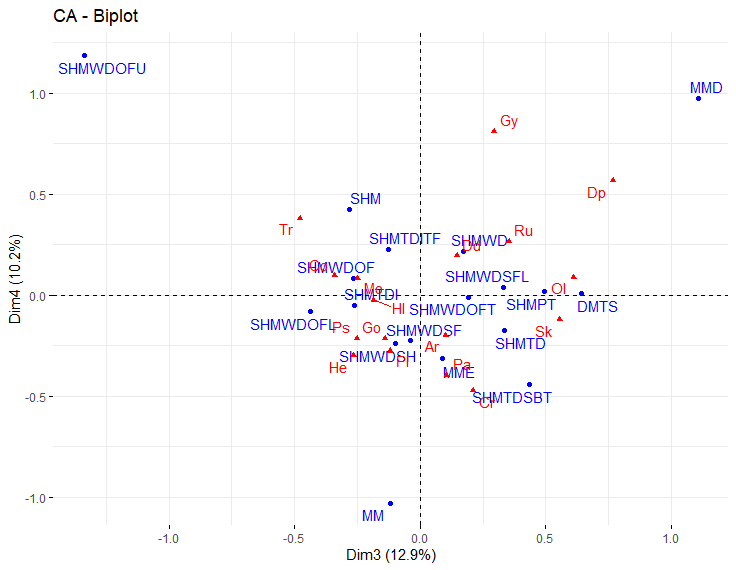


**Fig. S21**

Eigenvalue scree plot for the Cambrian Series 2 correspondence analysis. The eigenvalue for each component is plotted with the percentage of representative variance. Included in this figure is the Kaiser-Guttman criterion, recommending the retention of components 1 to 4.


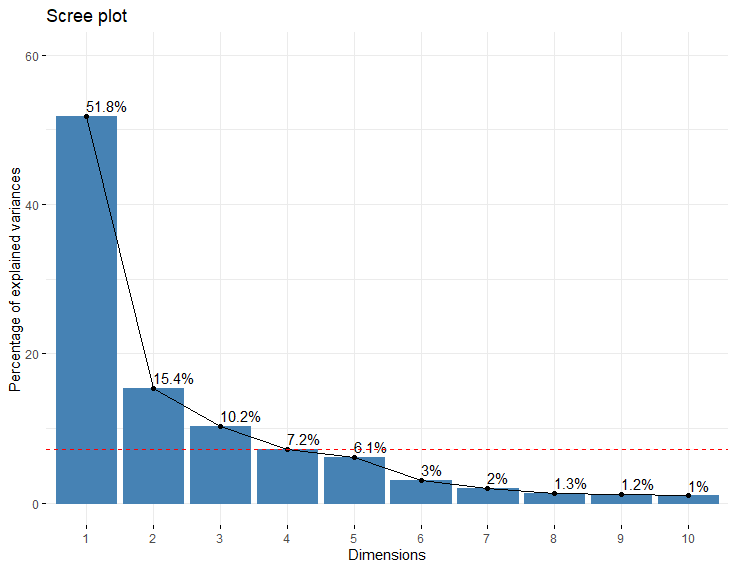


**Fig. S22**

Column (i.e., variable) contribution to the ordination of dimension 1 for the Cambrian Series 2. Red dashed line represents average expected value if data were random. Plot created in R with the factoextra package. Abbreviations provided in table S4 description.


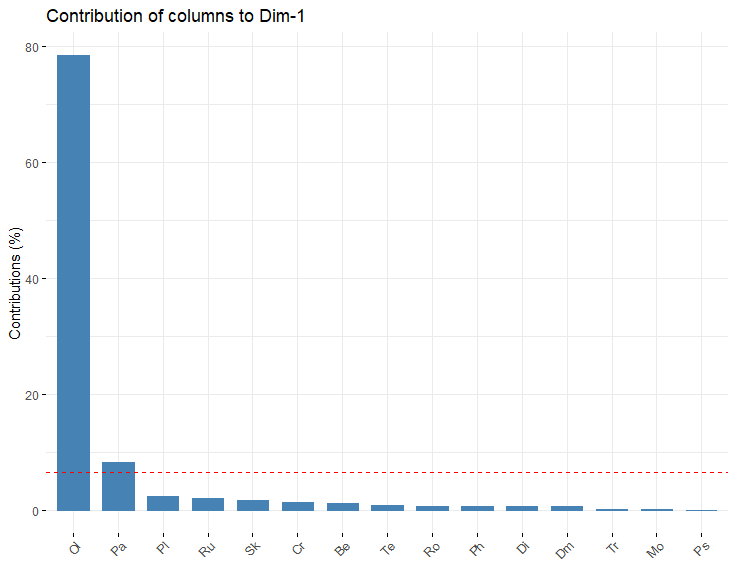


**Fig. S23**

Column (i.e., variable) contribution to the ordination of dimension 2 for the Cambrian Series 2. Red dashed line represents average expected value if data were random. Plot created in R with the factoextra package. Abbreviations provided in table S4 description.


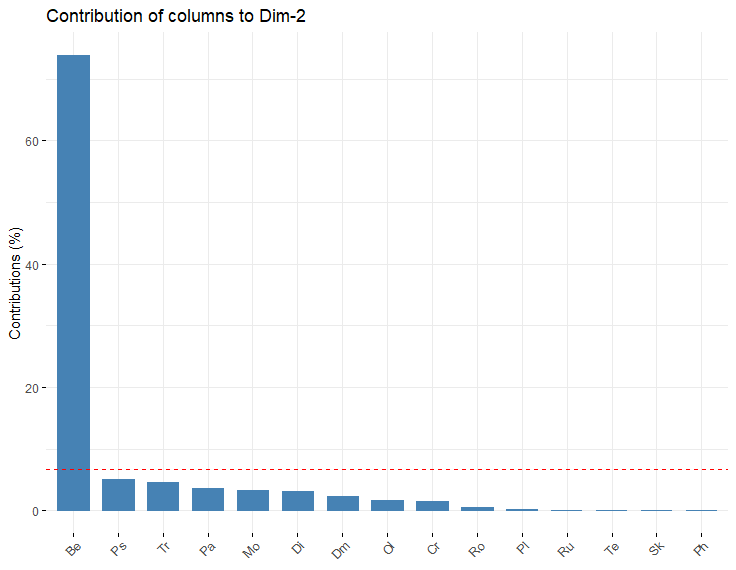


**Fig. S24**

Row (i.e., observation) contribution to the ordination of dimension 1 for the Cambrian Series 2. Red dashed line represents average expected value if data were random. Plot created in R with the factoextra package. Abbreviations provided in table S4 description.


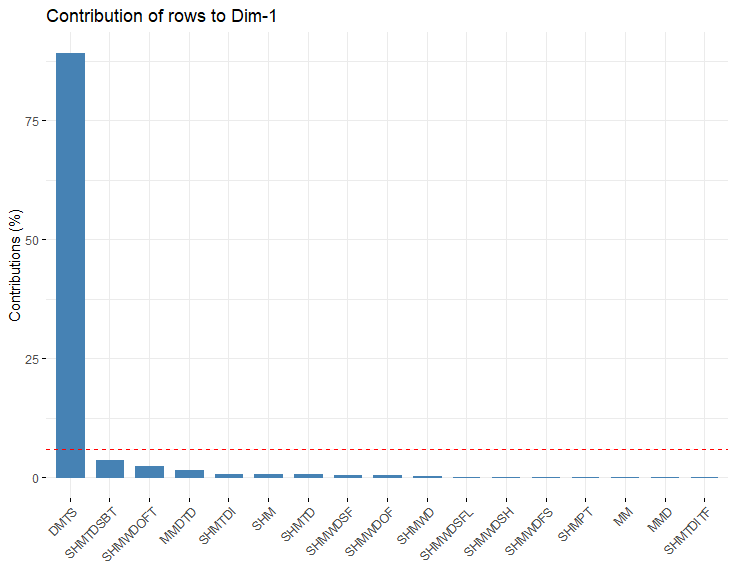


**Fig. S25**

Row (i.e., observation) contribution to the ordination of dimension 2 for the Cambrian Series 2. Red dashed line represents average expected value if data were random. Plot created in R with the factoextra package. Abbreviations provided in table S4 description.


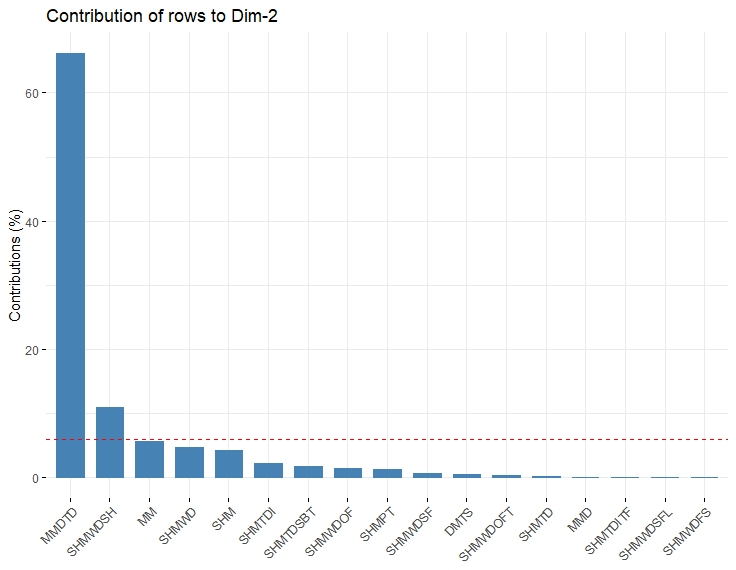


**Fig. S26**

Column (i.e., variable) quality of representation (cos2) for dimensions 1 and 2 of the Cambrian Series 2 ordination. Values of ≥0.66 are considered well represented, values ≥0.33 are considered moderately represented, and values <0.33 are considered poorly represented. Plot created in R with the factoextra package. Abbreviations provided in table S4 description.


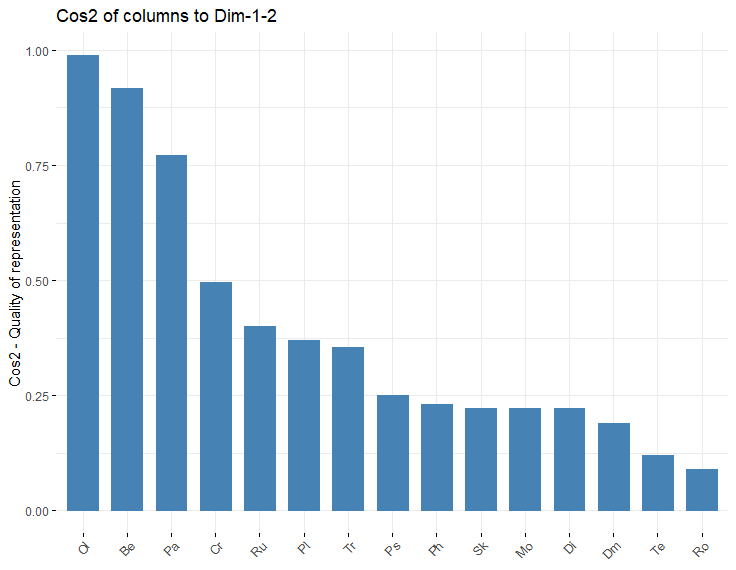


**Fig. S27**

Column (i.e., variable) quality of representation (cos2) for dimensions 3 and 4 of the Cambrian Series 2 ordination. Values of ≥0.66 are considered well represented, values ≥0.33 are considered moderately represented, and values <0.33 are considered poorly represented. Plot created in R with the factoextra package. Abbreviations provided in table S4 description.


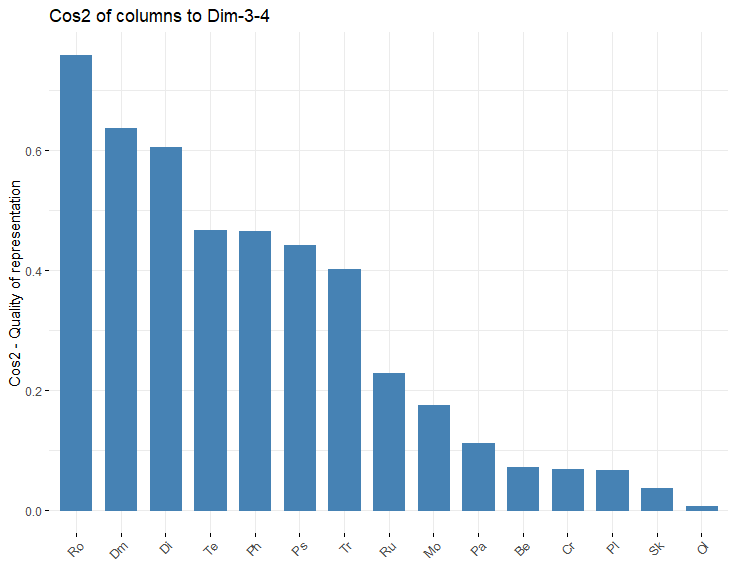


**Fig. S28**

Row (i.e., observation) quality of representation (cos2) for dimensions 1 and 2 of the Cambrian Series 2 ordination. Values of ≥0.66 are considered well represented, values ≥0.33 are considered moderately represented, and values <0.33 are considered poorly represented. Plot created in R with the factoextra package. Abbreviations provided in table S4 description.


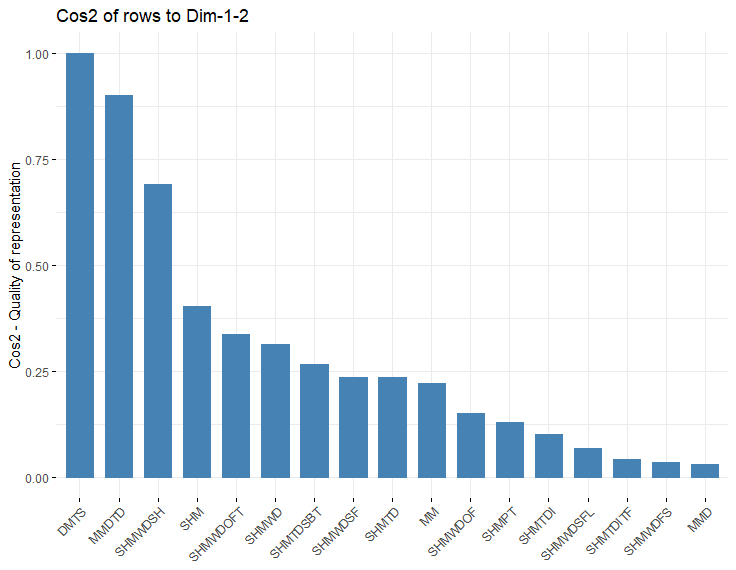


**Fig. S29**

Row (i.e., observation) quality of representation (cos2) for dimensions 3 and 4 of the Cambrian Series 2 ordination. Values of ≥0.66 are considered well represented, values ≥0.33 are considered moderately represented, and values <0.33 are considered poorly represented. Plot created in R with the factoextra package. Abbreviations provided in table S4 description.


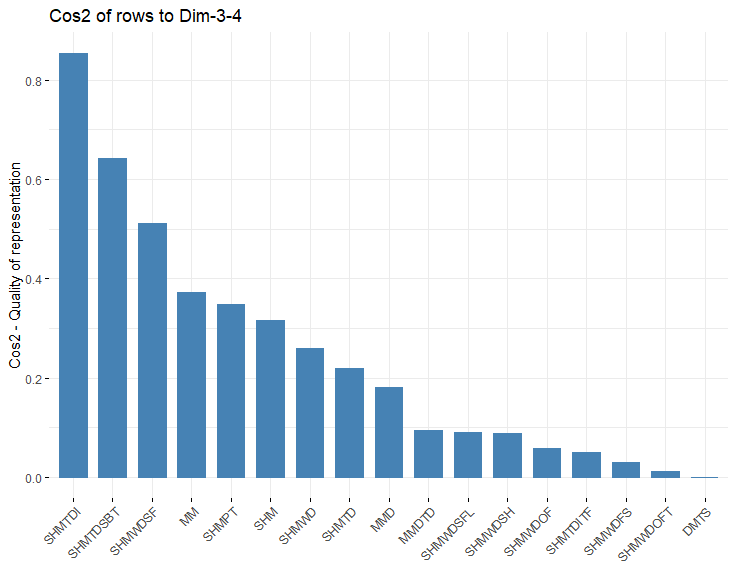


**Fig. S30**

Cambrian Series 2 biplot of Components 3 and 4. Plot created in R with the factoextra package. Abbreviations provided in table S4 description.


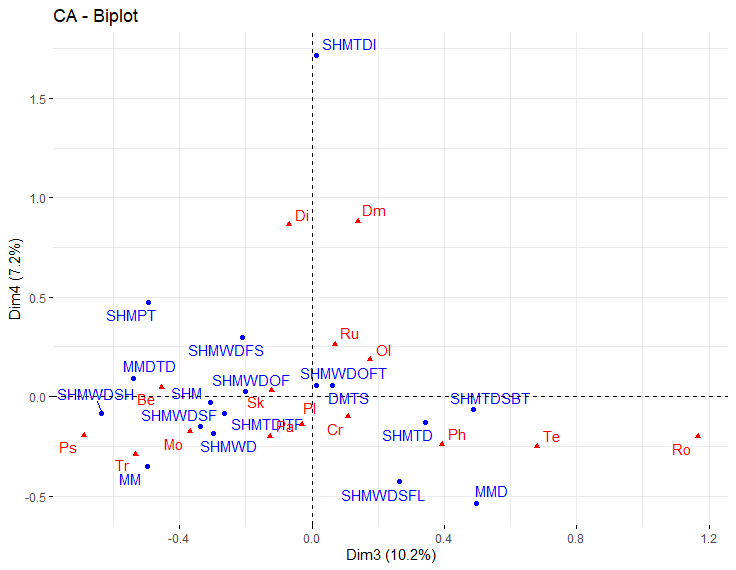


**R Code**

The following text was used in R Studio to conduct analysis and generate figures.

#CORRESPONCE ANLYSIS OF TRACE-FOSSIL DATA

require(FactoMineR)

require(factoextra)

require(corrplot)

#~~~~~~~~~~~~~~~~~~~~~~~~~~~~~~~~~~~

#EDIACARAN CORRESPONDENCE ANALYSIS

#~~~~~~~~~~~~~~~~~~~~~~~~~~~~~~~~~~~

EdData <- read.csv("~/R/EdData_ContingencyTable.csv", header=TRUE, row.names=1) #read in data from local files

EdData_Count <- sum(EdData) #sum of all values in contingency table

EdData_CA <- CA(EdData, graph=FALSE) #Correspondence Analysis

print(EdData_CA) #chi square: 242.1878, p=1.3e-11

chi2 <- 242.1878 #extracted from the print out command above

df <- (nrow(EdData) - 1) * (ncol(EdData) - 1) #calculate the degrees of freedom

ED_crtval <- qchisq(0.05, df, lower.tail=FALSE) #calculate the chi square critical value

#chi squared (df = 112, N = 143) = 242.19, p = 1.3e-11 > critical value (df = 112, α = 0.05) = 137.7 THEREFORE significant dependence between rows and columns

Ed_eig.val <- get_eigenvalue(EdData_CA)

Ed_dim <- 1/(9-1) #determine average column value if data were random -- 12.5%

fviz_screeplot(EdData_CA, addlabels=TRUE, ylim=c(0,40)) +

geom_hline(yintercept=12.5, linetype=2, color="red") #keep the first three dimensions

EdData_CA <- CA(EdData, ncp=3, graph=FALSE) #run CA again, only retaining the first three dimensions

fviz_ca_biplot(EdData_CA, repel=TRUE)

fviz_ca_biplot(EdData_CA, axes=c(1,3), repel=TRUE) #create second biplot with third dimension for supplementary files

#row analysis (depositional environment)

row <- get_ca_row(EdData_CA) #extract row [depositional environment] details for Ediacaran CA

fviz_ca_row(EdData_CA, col.row="cos2",

gradient.cols=c("grey","blue","red"),

alpha.row="cos2",

repel=TRUE) #visualize the quality of representation of rows [degree of association between row & axis - cos2]

corrplot(row$cos2, is.corr=FALSE)

fviz_cos2(EdData_CA, choice = "row", axes = 1:2) +

geom_hline(yintercept=0.66, linetype=2, color="green") +

geom_hline(yintercept=0.33, linetype=2, color="orange")

fviz_cos2(EdData_CA, choice = "row", axes = c(1,3))

fviz_ca_row(EdData_CA, col.row="contrib",

gradient.cols=c("#00AFBB","#E7B800","#FC4E07"),

alpha.row="contrib",

repel=TRUE) #visualize the individual rows contribution to the definition of dimensions

corrplot(row$contrib, is.corr=FALSE)

fviz_contrib(EdData_CA, choice="row", axes=1) #review contributions of rows to dim 1

fviz_contrib(EdData_CA, choice="row", axes=2) #review contributions of rows to dim 2

#column analysis (ichnogenera)

col <- get_ca_col(EdData_CA)

fviz_ca_col(EdData_CA, col.col="cos2",

gradient.cols=c("#00AFBB","#E7B800","#FC4E07"),

alpha.col="cos2",

repel=TRUE) #visualize the quality of representation of columns [degree of association between row & axis - cos2]

corrplot(col$cos2, is.corr=FALSE)

fviz_cos2(EdData_CA, choice = "col", axes = 1:2) +

geom_hline(yintercept=0.66, linetype=2, color="green") +

geom_hline(yintercept=0.33, linetype=2, color="orange")

fviz_cos2(EdData_CA, choice = "col", axes = c(1,3))

fviz_ca_col(EdData_CA, col.col="contrib",

gradient.cols=c("#00AFBB","#E7B800","#FC4E07"),

alpha.col="contrib",

repel=TRUE) #visualize the individual columns contribution to the definition of dimensions

corrplot(col$contrib, is.corr=FALSE)

fviz_contrib(EdData_CA, choice="col", axes=1) #review contributions of columns to dim 1

fviz_contrib(EdData_CA, choice="col", axes=2) #review contributions of columns to dim 2

#Asymmetric biplots

fviz_ca_biplot(EdData_CA, map="rowgreen", arrow=c(TRUE, FALSE), repel=TRUE) #contribution biplot (M.Greenacre)

fviz_ca_biplot(EdData_CA, map="colgreen", arrow=c(TRUE, FALSE), repel=TRUE) #contribution biplot (M.Greenacre)

fviz_ca_biplot(EdData_CA, map="rowprincipal", arrow=c(TRUE, TRUE), repel=TRUE) #rows in principal coords, cols in standard coords

fviz_ca_biplot(EdData_CA, map="colprincipal", arrow=c(TRUE, TRUE), repel=TRUE) #cols in principal coords, rows in standard coords

fviz_ca_biplot(EdData_CA, axes=c(1,3), map="rowgreen", arrow=c(TRUE, FALSE), repel=TRUE) #contribution biplot w/ dim 1 & 3 (M.Greenacre)

fviz_ca_biplot(EdData_CA, axes=c(1,3), map="colgreen", arrow=c(TRUE, FALSE), repel=TRUE) #contribution biplot w/ dim 1 & 3 (M.Greenacre)

#Export values for comparison with Coran -- extactly the same, but mirrored on axis 1

write.csv(EdData_CA$col$coord, file = "FactoMineR_columns_Ed.csv")

write.csv(EdData_CA$row$coord, file = "FactoMineR_rows_Ed.csv")

#~~~~~~~~~~~~~~~~~~~~~~~~~~~~~~~~~~~

#TERRENUVIAN CORRESPONDENCE ANALYSIS

#~~~~~~~~~~~~~~~~~~~~~~~~~~~~~~~~~~~

TerData <- read.csv("~/R/TerData_ContingencyTable.csv", header=TRUE, row.names=1)

TerData_Count <- sum(TerData) #sum of all values in contingency table

TerData_CA <- CA(TerData, graph=FALSE)

print(TerData_CA) #chi square:515.5166, p=2.8e-17

chi2 <- 515.5166 #extracted from the print out command above

df <- (nrow(TerData) - 1) * (ncol(TerData) - 1) #calculate the degrees of freedom

Ter_crtval <- qchisq(0.05, df, lower.tail=FALSE) #calculate the chi square critical value

#chi squared (df = 272, N = 521) = 515.52, p = 2.8e-17 > critical value (df = 272, α = 0.05) = 311.47 THEREFORE significant dependence between rows and columns

Ter_eig.val <- get_eigenvalue(TerData_CA)

Ter_dim <- 1/(17-1) #determine average column value if data were random -- 6.25%

fviz_screeplot(TerData_CA, addlabels=TRUE, ylim=c(0,40)) +

geom_hline(yintercept=6.25, linetype=2, color="red") #suggestion to keep 6 dims, keep the first 4 dims for ease of workflow

TerData_CA <- CA(TerData, ncp=4, graph=FALSE) #run CA again, only retaining the first four dimensions

fviz_ca_biplot(TerData_CA, repel=TRUE)

fviz_ca_biplot(TerData_CA, axes = c(3,4), repel=TRUE)

#row analysis (depositional environment)

row <- get_ca_row(TerData_CA) #extract row [depositional environment] details for Terrenuvian CA

fviz_ca_row(TerData_CA, col.row="cos2",

gradient.cols=c("#00AFBB","#E7B800","#FC4E07"),

alpha.row="cos2",

repel=TRUE) #visualize the quality of representation of rows [degree of association between row & axis - cos2]

fviz_ca_row(TerData_CA, axes = c(3,4), col.row="cos2",

gradient.cols=c("#00AFBB","#E7B800","#FC4E07"),

alpha.row="cos2",

repel=TRUE) #visualize the quality of representation of rows for Dim 3 & 4

fviz_cos2(TerData_CA, choice = "row", axes = 1:2)+

geom_hline(yintercept=0.66, linetype=2, color="green") +

geom_hline(yintercept=0.33, linetype=2, color="orange")

fviz_cos2(TerData_CA, choice = "row", axes = 3:4)

corrplot(row$cos2, is.corr=FALSE)

fviz_ca_row(TerData_CA, col.row="contrib",

gradient.cols=c("#00AFBB","#E7B800","#FC4E07"),

alpha.row="contrib",

repel=TRUE) #visualize the individual rows contribution to the definition of dimensions

corrplot(row$contrib, is.corr=FALSE)

fviz_contrib(TerData_CA, choice="row", axes=1) #review contributions of rows to dim 1

fviz_contrib(TerData_CA, choice="row", axes=2) #review contributions of rows to dim 2

#column analysis (ichnogenera)

col <- get_ca_col(TerData_CA)

fviz_ca_col(TerData_CA, col.col="cos2",

gradient.cols=c("#00AFBB","#E7B800","#FC4E07"),

alpha.col="cos2",

repel=TRUE) #visualize the quality of representation of columns [degree of association between row & axis - cos2]

fviz_cos2(TerData_CA, choice = "col", axes = 1:2)+

geom_hline(yintercept=0.66, linetype=2, color="green") +

geom_hline(yintercept=0.33, linetype=2, color="orange")

fviz_cos2(TerData_CA, choice = "col", axes = 3:4)

corrplot(col$cos2, is.corr=FALSE)

fviz_ca_col(TerData_CA, col.col="contrib",

gradient.cols=c("#00AFBB","#E7B800","#FC4E07"),

alpha.col="contrib",

repel=TRUE) #visualize the individual columns contribution to the definition of dimensions

corrplot(col$contrib, is.corr=FALSE)

fviz_contrib(TerData_CA, choice="col", axes=1) #review contributions of columns to dim 1

fviz_contrib(TerData_CA, choice="col", axes=2) #review contributions of columns to dim 2

#Asymmetric biplot

fviz_ca_biplot(TerData_CA, map="rowgreen", arrow=c(TRUE, FALSE), repel=TRUE) #contribution biplot (M.Greenacre)

fviz_ca_biplot(TerData_CA, map="colgreen", arrow=c(TRUE, FALSE), repel=TRUE) #contribution biplot (M.Greenacre)

#Export values for comparison with Coran -- extactly the same, but mirrored on axis 2

write.csv(TerData_CA$col$coord, file = "FactoMineR_columns_Ter.csv")

write.csv(TerData_CA$row$coord, file = "FactoMineR_rows_Ter.csv")

#~~~~~~~~~~~~~~~~~~~~~~~~~~~~~~~~~~~

#SERIES 2 CORRESPONDENCE ANALYSIS

#~~~~~~~~~~~~~~~~~~~~~~~~~~~~~~~~~~~

S2Data <- read.csv("~/R/S2Data_ContingencyTable.csv", header=TRUE, row.names=1)

S2Data_CA <- CA(S2Data, graph=FALSE)

print(S2Data_CA) #chi square:1460.166, p=3.8e-180

S2_eig.val <- get_eigenvalue(S2Data_CA)

S2_dim <- 1/(15-1) #determine average column value if data were random -- 7.14%

fviz_screeplot(S2Data_CA, addlabels=TRUE, ylim=c(0,60)) +

geom_hline(yintercept=7.14, linetype=2, color="red") #keep the first four dimensions

S2Data_CA <- CA(S2Data, ncp=4, graph=FALSE) #run CA again, only retaining the first four dimensions

fviz_ca_biplot(S2Data_CA, repel=TRUE)

fviz_ca_biplot(S2Data_CA, axes = c(3,4), repel=TRUE)

#row analysis (depositional environment)

row <- get_ca_row(S2Data_CA) #extract row [depositional environment] details for Series 2 CA

fviz_ca_row(S2Data_CA, col.row="cos2",

gradient.cols=c("#00AFBB","#E7B800","#FC4E07"),

alpha.row="cos2",

repel=TRUE) #visualize the quality of representation of rows [degree of association between row & axis - cos2]

corrplot(row$cos2, is.corr=FALSE)

fviz_cos2(S2Data_CA, choice = "row", axes = 1:2) +

geom_hline(yintercept=0.66, linetype=2, color="green") +

geom_hline(yintercept=0.33, linetype=2, color="orange")

fviz_cos2(S2Data_CA, choice = "row", axes = 3:4)

fviz_ca_row(S2Data_CA, col.row="contrib",

gradient.cols=c("#00AFBB","#E7B800","#FC4E07"),

alpha.row="contrib",

repel=TRUE) #visualize the individual rows contribution to the definition of dimensions

corrplot(row$contrib, is.corr=FALSE)

fviz_contrib(S2Data_CA, choice="row", axes=1) #review contributions of rows to dim 1

fviz_contrib(S2Data_CA, choice="row", axes=2) #review contributions of rows to dim 2

#column analysis (ichnogenera)

col <- get_ca_col(S2Data_CA)

fviz_ca_col(S2Data_CA, col.col="cos2",

gradient.cols=c("#00AFBB","#E7B800","#FC4E07"),

alpha.col="cos2",

repel=TRUE) #visualize the quality of representation of columns [degree of association between row & axis - cos2]

corrplot(col$cos2, is.corr=FALSE)

fviz_cos2(S2Data_CA, choice = "col", axes = 1:2) +

geom_hline(yintercept=0.66, linetype=2, color="green") +

geom_hline(yintercept=0.33, linetype=2, color="orange")

fviz_cos2(S2Data_CA, choice = "col", axes = 3:4)

fviz_ca_col(S2Data_CA, col.col="contrib",

gradient.cols=c("#00AFBB","#E7B800","#FC4E07"),

alpha.col="contrib",

repel=TRUE) #visualize the individual columns contribution to the definition of dimensions

corrplot(col$contrib, is.corr=FALSE)

fviz_contrib(S2Data_CA, choice="col", axes=1) #review contributions of columns to dim 1

fviz_contrib(S2Data_CA, choice="col", axes=2) #review contributions of columns to dim 2

#Asymmetric biplot

fviz_ca_biplot(S2Data_CA, map="rowgreen", arrow=c(TRUE, FALSE), repel=TRUE) #contribution biplot (M.Greenacre)

#Export values for comparison with Coran -- extactly the same, but mirrored on axis 1

write.csv(S2Data_CA$col$coord, file = "FactoMineR_columns_S2.csv")

write.csv(S2Data_CA$row$coord, file = "FactoMineR_rows_S2.csv")
